# Development and Evaluation of the Ancestry Informative Marker Panel of the VISAGE Basic Tool

**DOI:** 10.3390/genes12081284

**Published:** 2021-08-22

**Authors:** María de la Puente, Jorge Ruiz-Ramírez, Adrián Ambroa-Conde, Catarina Xavier, Jacobo Pardo-Seco, Jose Álvarez-Dios, Ana Freire-Aradas, Ana Mosquera-Miguel, Theresa E. Gross, Elaine Y. Y. Cheung, Wojciech Branicki, Michael Nothnagel, Walther Parson, Peter M. Schneider, Manfred Kayser, Ángel Carracedo, Maria Victoria Lareu, Christopher Phillips, on behalf of the VISAGE Consortium

**Affiliations:** 1 Forensic Genetics Unit, Institute of Forensic Sciences, University of Santiago de Compostela, 15782 Santiago de Compostela, Spain; m.delapuente.vila@gmail.com (M.d.l.P.); ruizramirez.jorge@gmail.com (J.R.-R.); ambroa.adrian@gmail.com (A.A.-C.); ana.freire3@hotmail.com (A.F.-A.); ana.mosquera@usc.es (A.M.-M.); angel.carracedo@usc.es (Á.C.); mvictoria.lareu@usc.es (M.V.L.); 2 Institute of Legal Medicine, Medical University of Innsbruck, 6020 Innsbruck, Austria; catarinagaxavier@hotmail.com (C.X.); walther.parson@i-med.ac.at (W.P.); 3 Genetics, Vaccines, Infectious Diseases and Pediatrics Research Group (GENVIP Group), Instituto de Investigación Sanitaria de Santiago de Compostela, 15706 Santiago de Compostela, Spain; j.pardoseco@gmail.com; 4 Faculty of Mathematics, University of Santiago de Compostela, 15705 Santiago de Compostela, Spain; joseantonio.alvarez.dios@usc.es; 5 Institute of Legal Medicine, Faculty of Medicine and University Clinic, University of Cologne, 50823 Cologne, Germany; gross.theresa@gmail.com (T.E.G.); elaine.yy.cheung@outlook.com (E.Y.Y.C.); peter.schneider@uk-koeln.de (P.M.S.); 6 Hessisches Landeskriminalamt, 65187 Wiesbaden, Germany; 7 Malopolska Centre of Biotechnology, Jagiellonian University, 30-387 Kraków, Poland; wojciech.branicki@uj.edu.pl; 8 Cologne Center for Genomics, University of Cologne, 50823 Cologne, Germany; mnothnag@ini-koeln.de; 9 University Hospital Cologne, 50937 Cologne, Germany; 10 Forensic Science Program, The Pennsylvania State University, University Park, State College, PA 16802, USA; 11 Department of Genetic Identification, Erasmus MC University Medical Center Rotterdam, 3015 CN Rotterdam, South Holland, The Netherlands; m.kayser@erasmusmc.nl; 12 Fundación Pública Galega de Medicina Xenómica (FPGMX), 15706 Santiago de Compostela, Spain

**Keywords:** bio-geographical ancestry, massively parallel sequencing, ancestry informative markers, SNPs, 1000 Genomes, Human Origins SNP array

## Abstract

We detail the development of the ancestry informative single nucleotide polymorphisms (SNPs) panel forming part of the VISAGE Basic Tool (BT), which combines 41 appearance predictive SNPs and 112 ancestry predictive SNPs (three SNPs shared between sets) in one massively parallel sequencing (MPS) multiplex, whereas blood-based age analysis using methylation markers is run in a parallel MPS analysis pipeline. The selection of SNPs for the BT ancestry panel focused on established forensic markers that already have a proven track record of good sequencing performance in MPS, and the overall SNP multiplex scale closely matched that of existing forensic MPS assays. SNPs were chosen to differentiate individuals from the five main continental population groups of Africa, Europe, East Asia, America, and Oceania, extended to include differentiation of individuals from South Asia. From analysis of 1000 Genomes and HGDP-CEPH samples from these six population groups, the BT ancestry panel was shown to have no classification error using the Bayes likelihood calculators of the *Snipper* online analysis portal. The differentiation power of the component ancestry SNPs of BT was balanced as far as possible to avoid bias in the estimation of co-ancestry proportions in individuals with admixed backgrounds. The balancing process led to very similar cumulative population-specific divergence values for Africa, Europe, America, and Oceania, with East Asia being slightly below average, and South Asia an outlier from the other groups. Comparisons were made of the African, European, and Native American estimated co-ancestry proportions in the six admixed 1000 Genomes populations, using the BT ancestry panel SNPs and 572,000 Affymetrix Human Origins array SNPs. Very similar co-ancestry proportions were observed down to a minimum value of 10%, below which, low-level co-ancestry was not always reliably detected by BT SNPs. The *Snipper* analysis portal provides a comprehensive population dataset for the BT ancestry panel SNPs, comprising a 520-sample standardised reference dataset; 3445 additional samples from 1000 Genomes, HGDP-CEPH, Simons Foundation and Estonian Biocentre genome diversity projects; and 167 samples of six populations from in-house genotyping of individuals from Middle East, North and East African regions complementing those of the sampling regimes of the other diversity projects.

## 1. Introduction

In the last ten years, forensic DNA analysis has been extended beyond identification tests that link a suspect to crime-scene evidence using STR profiling, into areas of genetic analysis designed to predict a range of physical characteristics of unidentified trace donors, mainly defined by single nucleotide polymorphisms (SNPs). Such SNP tests, applicable when no suspect has been identified by investigators, have been termed forensic DNA phenotyping (FDP), and until recently, consisted of tests to infer bio-geographical ancestry (BGA) [1], or to predict certain externally visible characteristics (EVCs) [2]. These SNP-based tests have now been supplemented with DNA analyses that measure the methylation status of carefully chosen CpG sites showing age-correlated methylation patterns in order to estimate a donor’s chronological age from the same DNA samples used for BGA and EVC tests [3]. The array of FDP tests now being adopted can serve to progress a major criminal investigation lacking strong leads, resurrect cold cases by providing new genetic data about old crime-scene DNA samples, and aid in the identification of the missing or historical remains [4]. While FDP tests have been gaining traction as supplementary analysis regimes available to forensic laboratories, the emergence of massively parallel sequencing (MPS) now provides major enhancements for forensic DNA analysis, including improved sensitivity to analyse minimal amounts of evidential material, larger multiplexes that can combine conventional identification STRs with SNPs for FDP analysis, and greater detail about the detectable variation within a sequence. The VISible Attributes through GEnomics (VISAGE) Consortium was initiated in 2017 to develop new MPS-based tools for predicting the BGA, appearance based on common EVCs, and age of an unidentified crime-scene DNA donor.

VISAGE adopted a two-step program to create MPS tools for FDP: developing a Basic Tool as a pilot assay, followed by the development of an Enhanced Tool with more markers and extended capabilities. The VISAGE Basic Tool (herein BT) is the first forensic MPS test combining markers for predicting eye, hair, and skin colour with those for BGA inference in a single assay, along with a saliva-blood focused age estimation assay requiring a parallel assay and workflow due to the bisulphite conversion steps needed to measure methylation. The VISAGE BT was founded on marker panels which have been shown to be informative for the three pigmentation traits [5,6,7] and for continental-scale population differentiation [8,9,10,11,12]. The level of multiplexing necessary to accommodate sufficient SNPs to yield optimum predictions of both EVCs and continental BGA was in part dictated by experience of building multiplexes for forensic MPS analysis [8,12,13]. Therefore, for the BT assay, which acts as a pilot test, we decided to design a moderate PCR scale of 150 to 160 SNPs by combining the 41 well-established HIrisPlex-S markers, which have been successfully applied in previous SNaPshot and MPS assays [5,6,7,13], with up to 120 ancestry-informative marker (AIM) SNPs. A panel of 120 AIM SNPs represents a reduced multiplex size compared to the one commercially available forensic ancestry panel for MPS, i.e., the Thermo Fisher Precision ID Ancestry Panel, comprising 165 AIMs [14], although similar in scale to another panel of 127 AIMs [8]. For this reason, we selected markers with the maximum possible ancestry informativeness for any given population, and then balanced these to ensure the major population groups had broadly comparable levels of differentiation. In contrast to the previous development of the 127-SNP Global AIMs panel (gAIMs [8]), South Asians, i.e., individuals from the Indian subcontinent, were treated as a separate population group.

This paper describes the compilation of component SNPs into the ancestry informative marker set forming the major portion of the VISAGE Basic Tool for appearance-ancestry. The development of the AIMs set and its evaluation involved four stages: i. Selection of markers and balancing of their relative population informativeness proportions in the multiplex to ensure comparable levels of continental population group differentiation; ii. Generation of a large-scale reference dataset built on open-access, genome-wide variant datasets, plus our own MPS genotyping of in-house population samples from which to apply the statistical analysis pipelines accompanying the BT assay; iii. Genotype concordance studies and an audit of existing genome-wide variant datasets for the selected AIM SNPs by analysing whole-genome sequence data released by the 1000 Genomes Project in 2020 [15,16,17]; and iv. Evaluation of the panel’s capacity to make efficient population differentiations using existing likelihood-based ancestry inference tests.

## 2. Materials and Methods

### 2.1. Selection and Balancing of Component Ancestry Informative SNPs

The BT assay design targeted an AIMs panel of approximately 120 SNPs, based on the fact that ~25% of the full multiplex would be occupied with the 41 markers of HIrisPlex-S, and shared use of key SNPs rs16891982 (in *SLC45A2* gene), rs1426654 (*SLC24A5*), rs12913832 (*HERC2*) for both ancestry and appearance predictive purposes. Because tri-allelic SNPs have been found to be useful for the detection of mixed DNA [18], but when carefully selected have also been informative for ancestry to comparable levels as binary SNPs [19], we expanded their number in the multiplex above those of the previous gAIMs panel (which had six tri-allelic SNPs of a total 127 loci [8]). Twenty tri-allelic SNPs showing the highest levels of population divergence were selected from a very large candidate pool created from a genome-wide compilation of this type of human variation [20].

Careful consideration was made to balance the differentiation of the five main continental population groups of sub-Saharan Africa, Europe, East Asia, Oceania and America (herein, AFR, EUR, EAS, OCE and AMR) with differentiation of the South Asian sub-group (SAS). We decided to target 60–70 SNPs for continental differentiation, plus 20 SNPs to distinguish South Asia from the neighbouring population groups of Europe and East Asia. 

SNPs for continental differentiation were compiled from four well-established AIM sets: i. the 127-SNP gAIMs ancestry panel; ii. the 446-SNP LACE (Latin American Cancer Epidemiology) ancestry panel [21]; iii–iv. the 165-SNP Thermo Fisher Precision ID Ancestry Panel (PIAP) [14], which includes the widely used 56-SNP Kiddlab ancestry panel [22]. SNPs differentiating South Asian and European populations were compiled from the above panels and three dedicated AIM sets for such geographic distinctions: v. the best European differentiating SNPs from the EUROFORGEN NAME panel [23]; vi. the Shriver group’s US admixture mapping panel, with many highly European-informative loci [24]; and vii. the *Eurasiaplex* South Asian informative panel [9]. 

The continental SNPs in BT were balanced in terms of their individual population differentiation power to equilibrate as far as possible the five continental groups, ensuring that no one population group had excessive numbers of indicative SNPs, which could potentially bias calculations of co-ancestry proportions in persons with admixed backgrounds (e.g., excessive numbers of African-informative SNPs in such a small panel could overestimate this co-ancestry compared to the others). For each SNP, this process records the population specific divergence (*I_n_*_AFR_; *I_n_*_EUR_; *I_n_*_E ASN_; etc., the metric divergence is capitalised and measures the degree of differentiation per marker) to guide the removal or addition of SNPs for a specific population comparison in order to improve overall *I_n_* balance. To reach this balance, the *Snipper* online ancestry analysis portal [25] was used to estimate cumulative *I_n_* values, as previously described for the development of the gAIMs panel [8]. As BT had a smaller number of SNPs than gAIMs, we did not expect to reach a comparably narrow range of finely balanced *I_n_* values. 

### 2.2. Compilation of a 4132-Sample Population Dataset

A comprehensive population dataset for the BT ancestry SNPs was generated by compiling online whole-genome-sequencing variant data from 3965 samples and adding in-house genotyping of 167 samples from six populations, chosen to cover geographic gaps in data for several under-represented regions, i.e., 30 from Eritrea, 16 from Somalia, 32 from Morocco, 32 from Central Iraq, 28 from the Kurdistan region of Iraq and 29 from Turkey (Turkish resident in Germany). An additional 277 DNA samples from the HGDP-CEPH human diversity panel were genotyped with the BT assay with the goal of further extending the geographic coverage of Oceanian, Native American and Middle East (herein ME) population variation, i.e., regions not covered by 1000 Genomes Phase 3 populations. However, the 1000 Genomes Consortium completed whole-genome-sequencing of the full HGDP-CEPH panel in 2019 soon after our in-house genotyping had been completed, so we compiled the complete dataset from 1000 Genomes and used the overlapping sample data to measure the MPS genotyping concordance of BT. This was accomplished for the whole marker set of BT (i.e., including the 41 appearance markers) to gauge sequencing performance across the complete multiplex. 

Of the total 4132 SNP genotype profiles compiled, 520 were used as a standardised six population group reference dataset for uploading as training sets to the *Snipper* ancestry analysis portal, with the aim of providing the closest possible equivalence of sample sizes amongst the continental and South Asian regions. The standardised reference dataset consisted of: sub-Saharan Africans represented by 108 1000 Genomes 30X sequence coverage (1KG-30) Yoruba from Nigeria (YRI); Europeans by 99 1KG-30 CEPH Europeans from Utah (CEU); East Asians by 103 1KG-30 Han Chinese from Beijing (CHB); South Asians by 103 1KG-30 Gujarati from Houston (GIH); Oceanians by 28 HGDP-CEPH Papuans from Bougainvillea and Papua New Guinea; Native Americans by 79 samples, comprising 61 HGDP-CEPH samples from Maya, Pima populations, Colombians and Amazonian Surui and Karitiana populations, supplemented by 18 1KG-30 Peruvians from Lima, Peru (PEL) which we had previously analysed and showed no detectable non-American co-ancestry (in-silico analysis of 572,743 Affymetrix Human Origins SNPs, see Table 10.5 of [26]).

Although we established the above standardised reference dataset, the additional populations compiled can be flexibly included in *Snipper* or can replace those in the reference set at the user’s discretion by adjusting the Excel-based training set file uploaded to the ‘multiple profiles’ classifier portal (http://mathgene.usc.es/snipper/analysismultipleprofiles.html, accessed on 20 August 2021), which generates a Bayes likelihood ratio statistical test for ancestry and a matched 2D principal component analysis (PCA) plot. For maximum flexibility, the other population data was arranged into a series of worksheets in the same core Excel reference file, allowing the end-user to select alternative reference populations from 1000 Genomes or HGDP-CEPH (e.g., HGDP-CEPH Pakistani South Asian samples rather than 1KG-30 Gujarati, or other populations from the Indian sub-continent). The same user-driven options apply to the VISAGE in-house population samples provided in a separate worksheet. The admixed populations in 1000 Genomes, comprising 96 African Caribbean individuals in Barbados (ACB), 61 Americans of African Ancestry in SW USA (ASW), 64 individuals with Mexican Ancestry from Los Angeles USA (MXL,) 94 Colombians from Medellin, Colombia (CLM), 104 Puerto Ricans from Puerto Rico (PUR), 67 of 85 PEL (i.e., with detected co-ancestry) were also compiled in a separate worksheet but would not be expected to be useful as training set data from which representative allele frequency estimates could be obtained, given their high levels of individual admixture.

Genome-wide variant data from Simons Foundation human genome diversity project (herein SGDP) has been available for several years [27], and offers additional information on geographic areas outside of those covered by 1000 Genomes or the HGDP-CEPH sampling regimes, in particular, Northeast Asia (broadly, Eastern Siberia east towards the Bering Straits); Southeast Island Asia; and Central South Asia (broadly, the Caucasus’ east towards the central Asian Steppe immediately north of South Asia). There are 278 genome-wide SNP datasets, of which 22 overlap with 1000 Genomes and 126 overlap with the HGDP-CEPH panel samples, leaving 130 unique samples, from 67 populations. The small sample size of SGDP highlights the very limited number of 1, 2 or 3 samples per region. Nevertheless, we compiled the 130 SGDP-unique BT AIM profiles into a dedicated worksheet and completed population analyses using the standardised reference set.

Genome-wide variant data from the Estonian Biocentre genome diversity project (herein, EGDP) largely mirrors the sampling regimes of SGDP [28], with a maximum of 16 samples per region, but mainly 1, 2 or 3 per region. EGDP has 402 whole-genome datasets from the sampling of 126 populations that almost all complement those of SGDP and provides extensive coverage of regions in Siberia, Northeast Asia, Eastern Europe, and Central South Asia. As with SGDP, the 402 EGDP BT AIM profiles were compiled into a dedicated worksheet and population analyses made with the standardised reference set. Note that the tri-allelic SNPs forming part of the BT AIMs panel are not reported by EGDP and an additional four ancestry SNPs do not have genotypes, so data completeness is ~83.5% (96/115 SNPs).

### 2.3. Genotyping Concordance amongst MPS Sequence Data and Online Databases

Genotyping concordance for the BT SNPs was assessed in two ways. First, a direct comparison was made between the MPS genotypes generated during developmental runs genotyping 277 HGDP-CEPH DNA samples for all 153 SNPs in BT, and the SNP genotypes reported by 1000 Genomes for the same DNA samples [16], providing 42,381 comparisons. Second, the genotypes listed by 1000 Genomes Phase 3 database for 2504 worldwide population samples (and available in the Ensembl browser at: http://www.ensembl.org/Homo_sapiens/Info/Index, accessed on 20 August 2021) were compared to the high coverage re-sequencing dataset for the same samples, released in early 2020 by the New York Genome Centre [17]. The high sequence coverage genotypes correspond to an average 30X coverage, while the current Phase 3 genotypes were produced from an average 2-3X coverage. We considered the high coverage data to represent more reliable genotype calls for the SNPs of BT, so these were compiled as the major part of the reference population data we used for statistical analysis of ancestry, as described above. 

The Coriell control DNA samples originally used as the genotyping concordance standards in the inter-laboratory evaluations of the BT assay [29] were also run as positive controls on each sequencing run performed for the in-house population studies.

### 2.4. Evaluation of Ancestry and Co-Ancestry Inference Efficiency of the BT AIMs

The efficiency of the BT AIMs to infer ancestry was assessed for a six-group differentiation of African, European, East Asian, South Asian, American and Oceanian ancestries. To measure classification error, one-out cross-validation of the standardised reference dataset of 520 samples was performed using the verbose cross validation option in *Snipper* (http://mathgene.usc.es/Snipper/analysispopfile2_new.html, accessed on 20 August 2021). For all other samples, STRUCTURE, PCA and neighbour joining tree (NJT) analyses were performed on each of the separate population sets, comprising admixed and unadmixed 1000 Genomes samples not used as reference data; HGDP-CEPH samples not used as reference data (i.e., no Oceanian, American population data); SGDP samples, with the six in-house populations included in the same analysis run; and EGDP samples (for 100 binary SNPs of 115 ancestry SNPs in BT). STRUCTURE v.2.3.4 [30] was used for runs consisting of five iterations per K (genetic cluster values K:2 to K:8 evaluated) consisting of 100,000 burnin steps and 100,000 MCMC steps, using correlated allele frequencies under the Admixture model. The optimum ‘K’ cluster number was inferred by plotting log probability of K and magnitude of ΔK, following the analyses of Evanno et al. [31]. Allele frequencies were updated only using individuals marked POPFLAG = 1 at the optimum genetic cluster number of K:6. Cluster membership proportion plots were constructed with CLUMPAK v.1.1 [32]. Multi-dimensional scaling (MDS) analysis and construction of neighbour-joining trees was implemented using R software v.3.5.0 [33] over an allele-distance matrix computed with the *pegas* R package [34].

The reliable identification of co-ancestry in DNA donors with admixed backgrounds can be considered important information that can be reported to investigators. Although there is likely to be some imprecision in the estimation of co-ancestry ratios when using relatively small-scale AIMs panels like BT, if the markers have been carefully balanced, they are more likely to be free of bias towards one particular co-ancestral population group and therefore match more closely the actual co-ancestry proportions in such individuals. To measure the reliability of co-ancestry estimation from identifying genetic clusters using the BT AIMs, we ran all 504 admixed 1000 Genomes samples described above (including all 85 PEL), together with the unadmixed populations from AFR, EUR, AMR and EAS, to keep the identified genetic clusters simple (K:4). The ACB and ASW samples represent typical African Caribbean and African American co-ancestry patterns of majority African co-ancestry and 0–30% European co-ancestry. The MXL, CLB, PUR and PEL samples represent more complex three-way co-ancestry patterns of major European co-ancestry, with Native American and African co-ancestries at varied ratios, with a few PEL individuals showing detectable East Asian co-ancestry [15]. We compared the BT data to the co-ancestry ratio estimates from the detailed genetic cluster analysis of the same 1000 Genomes-CEPH populations using 572,743 Affymetrix Human Origins SNPs (see Section 2.2. Although the parallel analyses with the Human Origins SNPs used the ADMIXTURE algorithm [35] to identify clusters, because data input for this algorithm uses only binary variant data, we applied STRUCTURE to ensure the tri-allelic SNP genotypes were assessed. Co-ancestry ratios were grouped into 10-percentiles (i.e., bins of one tenth of the samples from any one population) and the mean ratios of each of these 10-percentile groups in both analyses were assessed for goodness-of-fit using linear regression. Finally, we included a comparison with the genetic distance algorithm (GDA) co-ancestry estimator available in *Snipper*, developed from the work of Cheung et al. 2017 [36]. 

### 2.5. Evaluation of Ancestry and Co-Ancestry Inference Efficiency of the BT AIMs

The current gnomAD version 3.1 genome database compiles variation in almost 100,000 individuals from ten populations, so it provides a very efficient system for the detection of rare variation which could be potentially population informative. We screened the gnomAD 3.1 database to make a genomic audit of rare variations associated with the selected BT ancestry SNPs, comprising two types: i. rare, but potentially informative ‘flanking’ SNPs present on the same amplified fragment as the target SNP; and ii. additional polymorphic alleles in the binary ancestry SNPs, in the form of a third nucleotide substitution—creating a tri-allelic SNP where the allele-3 is either very rare or linked to a specific population group and hitherto undetected. Cataloguing rare third nucleotide substitutions is an important MPS quality control step, as it forewarns the user that the detected nucleotide at the target site is not a spurious sequencing result, but has been detected as a low frequency allele. We confined out searches for flanking SNPs within an arbitrary +/− 25 nucleotides of the target SNP position.

## 3. Results and Discussion

### 3.1. Ancestry Informative SNPs in BT

One hundred binary SNPs were compiled into the final BT ancestry panel, comprising 7 SNPs informative for sub-Saharan African populations, 15 European, 16 East Asian, 20 South Asian, 13 Oceanian and 17 American populations. The extra 12 ancestry SNPs of the total 100 loci were selected as they showed a high level of differentiation between European and other Eurasian population groups, namely East and South Asian and Middle Eastern populations. Table 1 outlines the source of each of these binary SNPs from the previous forensic AIMs panel studies described in Section 2.1 [8,9,21,22,23,24], with just one OCE-informative SNP added from a separate study that developed the *Pacifiplex* panel [11]. Over a quarter of SNPs (28 of 100) have been selected as forensic AIMs in multiple panels. 

**Figure 1:**
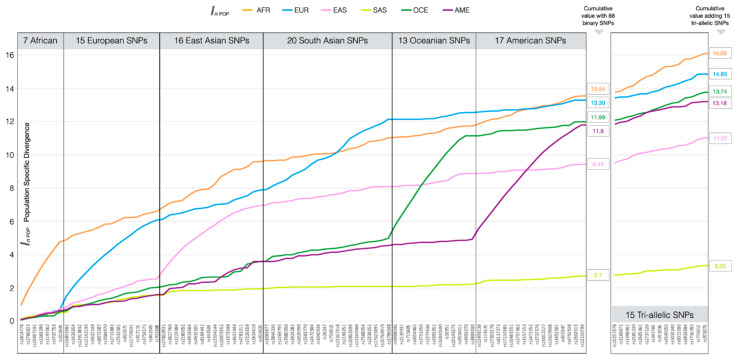
Accumulating population specific divergence values for each of the main population groups and the six sets of SNPs informative for their differentiation. The cumulative values are shown obtained from 88 binary SNPs (i.e., excluding 12 Eurasian-informative SNPs) and from the addition of the 15 tri-allelic SNPs in BT (VISAGE Basic Tool).

Balancing population specific divergence (PSD) values (excluding the 12 Eurasian divergent SNPs) was successful to a large extent and the cumulative PSD values for each of the six population groups are shown in Figure 1 (individual values per SNP in Table S1). AFR, EUR, OCE and AMR were well balanced with cumulative PSD values of 13.54, 13.30, 11.99 and 11.8, respectively. EAS with a cumulative value of 9.43 did not have enough informative SNPs to reach these levels of divergence, while SAS, with much less differentiation from EUR and EAS, only reached a cumulative value of 2.7. It is noteworthy that two BT appearance SNPs, rs6437783 (in *MYH15* gene) and rs1800414 (*OCA2*), shown in Table 1, had been selected for previous forensic ancestry panels as an EAS-informative SNP in gAIMs and Kiddlab AIM sets, respectively. Given the lower cumulative PSD value for EAS compared to other population groups, these two markers would be worth considering as joint ancestry-appearance SNPs along with the three already used in this way. 

From a candidate list of 20 tri-allelic SNPs, rs11238577, rs11990024, rs9845503, rs7853487 and rs809540 were rejected from the assay design, bringing the total number of AIMs in BT to 115, including the three shared appearance SNPs. When the 15 tri-allelic SNPs were added to the cumulative PSD plot of Figure 1 the balance was marginally improved for EAS with a cumulative PSD value of 11.01 closer to the average value for the other population groups.

Allele frequency estimates of the 115 BT AIMs based on the standardised reference grid outlined in Section 2.2 (YRI, CEU, CHB, GIH, plus HGDP-CEPH Americans and Oceanians) are given in File S1. Middle East allele frequency estimates are included based on 134 HGDP-CEPH samples from the Israeli Bedouin, Druze and Palestinian populations. Figure S1A–G summarise in simple bar charts the allelic variation in each binary AIM, arranged in sets of the AIMs informative for each population group and ranked by a simple delta allele frequency differential calculation comparing the informative-allele frequency in each target population against the average frequency in the other populations (except SAS-informative SNPs, ranked by SAS-EUR delta values). Figure S1G shows the allele frequencies of 12 Eurasian-informative SNPs in the relevant population groups. For comparison purposes we included North African (NAF) allele frequency estimates based on HGDP-CEPH Mozabite from Algeria, and delta values were estimated for EUR compared to a NAF-ME-SAS average).

### 3.2. Compilation of Reference and Test Population Datasets

File S2 contains all the Excel worksheets necessary for generating Bayes likelihood calculations and linked PCA analyses by uploading the BT ancestry SNP genotype data to *Snipper*. The active worksheet of reference and query SNP profiles uploaded to *Snipper* is in position 1 in Excel and has the label ‘1’ applied to all reference population profiles in the rightmost column, and ‘0’ to unknown profiles such as those compiled from a forensic sample. The values in cells A1, B1, C1 provide numbers of reference/query profiles, markers, and populations in the worksheet, respectively. All other SNP profiles compiled in the six supporting worksheets can be treated as test samples (i.e., marked with a ’0’), or used as alternative or additional reference population datasets (e.g., the HGDP-CEPH or VISAGE in-house ME population genotypes can be added to the reference profiles as a seventh population group).

### 3.3. Genotyping Concordance

#### 3.3.1. HGDP-CEPH in-House BT Genotypes vs. 1000 Genomes Whole-Genome-Sequence Data

File S3A lists all the pairwise genotype comparisons between in-house data for HGDP-CEPH samples and the data from 1000 Genomes published in 2020 [16]. This file examines all SNPs in BT except for one appearance marker, rs796296176, which did not have genotype data from the in-house analyses of HGDP-CEPH samples. Five HGDP-CEPH Middle East samples had excessive numbers of data gaps from in-house sequencing and were not included in genotype comparisons. Of the 153 total markers in BT, two ancestry SNPs had higher than average discordancy rates, indicating a problem with genotyping either from 1000 Genomes sequence analysis, or the BT MPS assay. First, tri-allelic SNP rs2737126 had 15/277 (5.4%) genotype discordancy, seemingly arising from failure to detect a G allele, when present as a GT or CG heterozygote, but not for all such genotypes. This phenomenon was not explained by detailed scrutiny of sequence data at this variant site. Second, the binary SNP rs3804030 had a disproportionately high genotype discordancy rate of 29/277 (10.47%), which appeared to be due to a failure to detect the rare, OCE-indicative C allele outside of Oceanian populations examined by 1000 Genomes. Interestingly, no discordances were seen for this SNP amongst pairwise comparisons of 27 HGDP-CEPH Oceanians.

File S3A shows that for the 151 SNPs of BT without apparent problems, the genotyping concordance was very high, reaching 99.96% concordance with 17 discordances largely appearing at random across the data grid, rather than systematically as detected in the above two SNPs. This high level of genotype concordance between the BT assay developed vs. 1000 Genomes whole genome sequence-based variant detection indicates the robustness of the MPS method and the BT multiplex developed by VISAGE. The no-call rate (unreported genotypes) was also low at 0.2% for 1000 Genomes, and 0.6% for our MPS genotyping.

#### 3.3.2. 1000 Genomes Phase 3 Genotypes vs. 1000 Genomes High Sequence Coverage Genotypes

**Figure 2:**
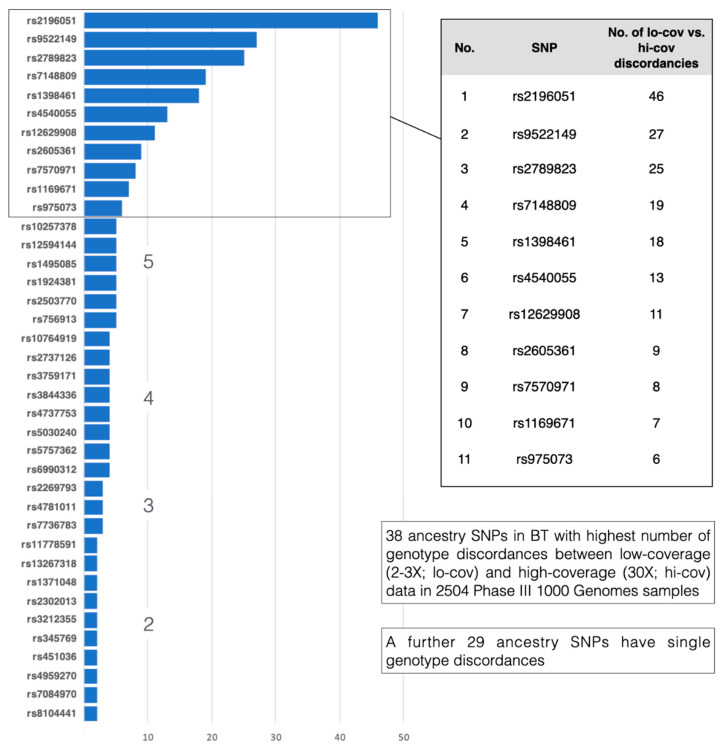
The 38 BT ancestry SNPs with the highest numbers of discordant genotypes between 1000 Genomes low coverage vs. high coverage sequence data. An additional 29 had only one discordant genotype. The full set of genotype comparisons for all BT SNPs and all 1000 Genomes samples are given in File S3B.

File S3B gives the pairwise comparisons for all BT SNPs between the current 1000 Genomes Phase 3 genotypes listed online by Ensembl, and the genotypes published following high coverage sequence analysis of the same samples by the New York Genome Centre [17]. As we have relied extensively on the online genotype data produced by 1000 Genomes Phase 3 for several years, the cross-checks of genotype calls comparing 30X and 2-3X sequence coverage that this concordance analysis provides are important. Amongst all 153 BT SNPs, 73 had zero genotype discordances and 66 showed largely inconsequential discordances numbering 1–5 genotype differences between each sequencing dataset (a locus-specific concordance of 99.96–99.8%). However, 14 SNPs had higher levels of six or more discordances, comprising 11 ancestry SNPs. Three ancestry SNPs: rs2789823, rs9522149 and rs2196051 had concordance rates ≤ 98.2% due to 25, 27 and 46 discordances, respectively. The BT ancestry SNPs genotype discordances of two or more in number are summarised in Figure 2. It can be assumed that higher sequence coverage provides more secure genotyping for a small, but significant proportion of SNPs in the 1000 Genomes variant database, of which 14 with higher-than-average levels of discordancy are part of BT. For this reason, all reference population data compiled from 1000 Genomes for VISAGE ancestry analyses with BT are based on the high coverage (30X) genotype datasets, which have yet to be made available in the Ensemble variant browser for 1000 Genomes.

### 3.4. Ancestry Inference Efficiency of the BT Ancestry SNPs

One-out cross validation of the reference dataset gave 100% correct classification in all cases, i.e., all 520 reference profiles were successfully assigned to their true population of origin. The likelihood ratio values produced by cross validation were consistently high and most samples gave likelihoods greater than ‘The profile is more than 1 billion times more likely to be from the true population of origin than another population’ (data not shown). 

**Figure 3:**
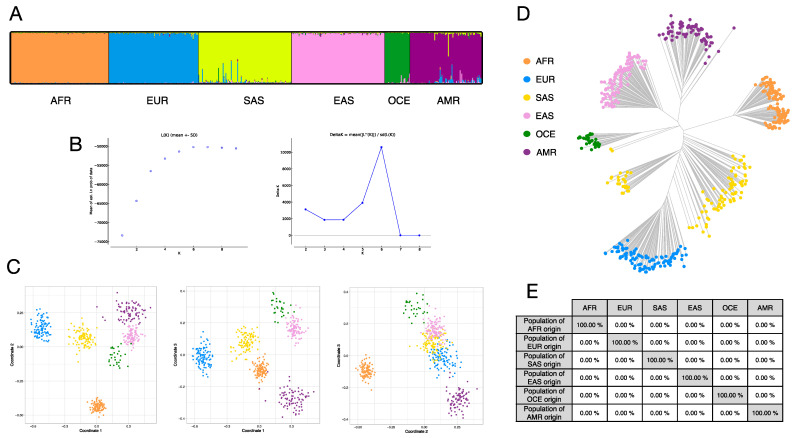
Ancestry analysis with 115 BT ancestry SNPs of the six populations of the standardised reference dataset (AFR: brown; EUR: blue; SAS: yellow; EAS: pink; OCE: green; AMR: purple), with consistent colours across three statistical analyses of A, C and D. (**A**) STRUCTURE cluster membership proportions at K = 6, indicated to be the optimum K number of genetic clusters by: (**B**) the mean L(K) (log probability of data) and ΔK plots from STRUCTURE runs, following the analyses of Evanno et al. [31]. (**C**) Multi-dimensional scaling (MDS) analysis showing principal component (PC) 1 vs. PC2 coordinates, PC1 vs. PC3 and PC2 vs. PC3 two-dimensional plots. (**D**) Neighbour joining tree (NJT) analysis. (**E**) Summary classification success table of cross validation of the standardised reference set.

Figure 3A shows the STRUCTURE analysis K:6 cluster plots for the reference populations (1–6) in worksheet 1 of File S1. Figure 3B shows the accompanying probability of data and Delta K statistical analyses indicating an optimum cluster number of 6 for these analyses. Figure 3C shows colour-matched MDS plots for the same samples for coordinate 1 vs. 2 (left plot), 1 vs. 3 (mid) and 2 vs. 3. Only a small degree of overlap between the OCE and EAS MDS clusters-of-points remains when comparing all three coordinate plots. Figure 3D shows the Neighbour Joining Tree (NJT) distance matrix results, which indicate a clear arrangement of branch lengths and spatial separation, apart from two SAS outliers close to the OCE set. The summary of cross-validation classification results in Figure 3E completes the ancestry analysis of these sample sets. 

Figure S2.1 summarises the same type of population analyses as Figure 3 applied to unadmixed (8–11, 14–25) and admixed (12–13, 26–29) 1000 Genomes samples (30X coverage genotypes) analysed against the reference population set. In nearly all cases, unadmixed samples show low levels of joint cluster membership. The admixed African populations give similar patterns to those found in other studies (but see Section 3.5) with European co-ancestry ranging from 5–30% K2 cluster membership. The admixed American samples from 1000 Genomes also show the expected proportions from K:2 and K:6 in the STRUCTURE analysis, with K:1 cluster membership forming the third African co-ancestry component, most evident in Puerto Ricans. The carefully curated Peruvian reference Native American samples are clearly discernible on the left of the columns of the K6 cluster. Equally consistently, admixed samples occupy the middle ground between the MDS clusters-of-points formed by the unadmixed samples, depending on the degree of their separation in coordinates 1 vs. 3 or 1 vs. 2 MDS plots, particularly the admixed Africans in 1 vs. 2.

Figure S2.2–S2.4 show identical analysis output and using the same reference populations for HGDP-CEPH samples, SGDP-VISAGE in-house samples and EGDP samples, respectively. The HGDP-CEPH samples show a degree of joint cluster membership proportions in regions whose populations occupy continental margins, e.g., Mozabite show African-European co-ancestry patterns (joint membership proportions) and Pakistani populations show some European co-ancestry. The SGDP and EGDP sample analyses are the most varied, reflecting the more widespread sampling locations and emphasis on continental margin regions such as Central South Asia. The SGDP-EGDP Oceanian and American samples give clear, unequivocal cluster memberships, underlining the careful choice of informative SNPs for these population groups. However, it should be noted that BT and all other equivalent MPS ancestry panels show Oceanian ancestry to be only a small proportion of the STRUCTURE cluster patterns in the single SGDP Maori and Hawaiian samples, indicating that AIMs selected from analysis of Melanesians (i.e., HGDP-CEPH Papuans) are not sufficiently informative to infer Polynesian ancestry.

### 3.5. Co-Ancestry Analysis of Known Admixed Individuals in 1000 Genomes with BT Ancestry SNPs 

#### 3.5.1. Comparison of Co-Ancestry Patterns Obtained with the Human Origins Panel and BT

**Figure 4:**
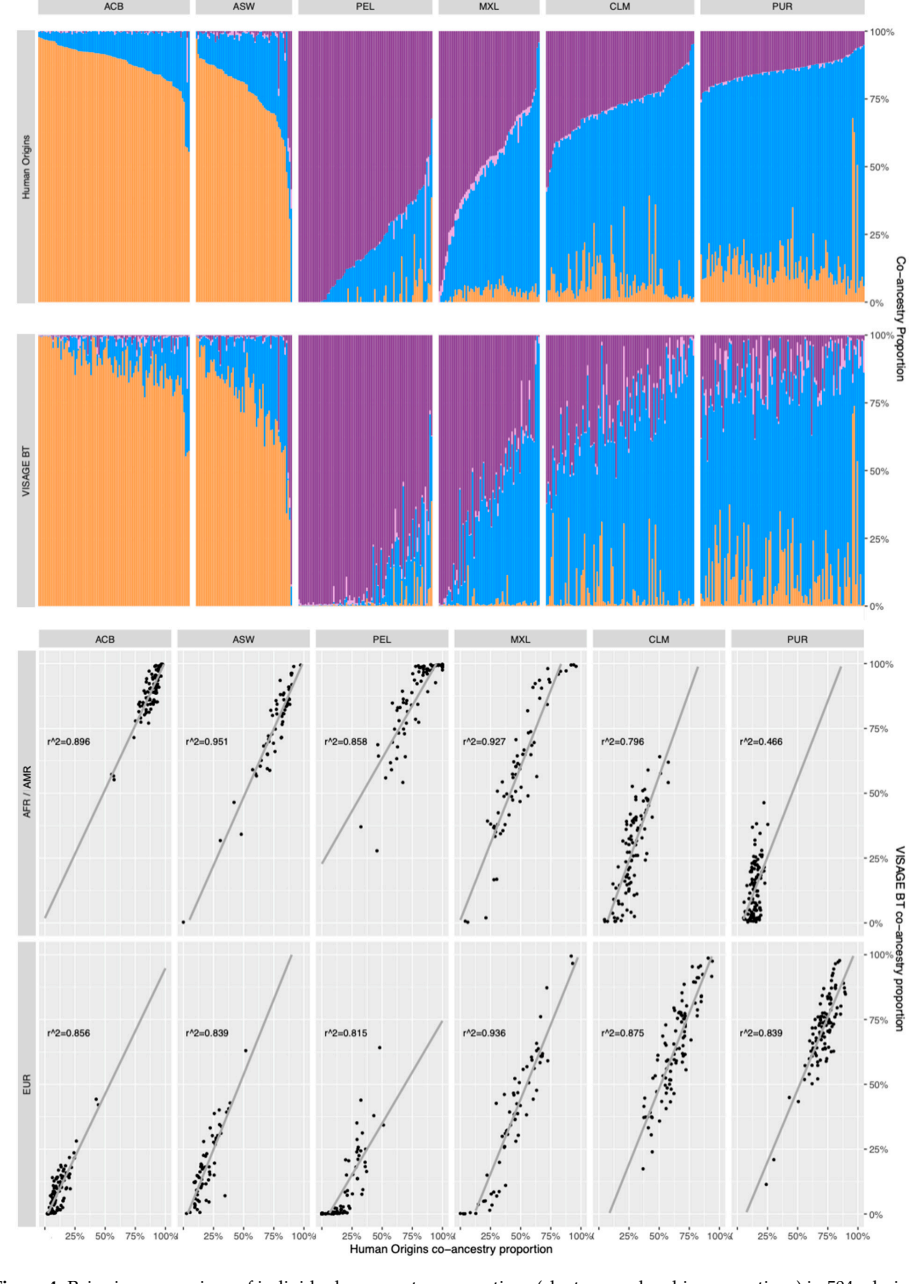
Pairwise comparison of individual co-ancestry proportions (cluster membership proportions) in 504 admixed samples from 1000 Genomes, analysed using the standardised reference set with Human Origins array, comprising >572,000 SNPs (using ADMIXTURE) and 115 VISAGE BT ancestry SNPs (using STRUCTURE). Co-ancestry proportions in four genetic clusters representing, African, European, Native American and East Asian, ancestries are given in File S4. The r^2^ correlation analysis plots of each admixed population group are shown below the corresponding cluster patterns.

Figure 4 shows a direct pairwise comparison of the co-ancestry patterns (cluster membership proportions) of 504 admixed samples from 1000 Genomes (see File S1C), analysed in combination with the standardised reference set with the >572,000 Human Origins array SNPs (using ADMIXTURE due to SNP data density) and the 115 ancestry SNPs of BT (using STRUCTURE). File S4 gives the genetic cluster proportions from both analyses on which the Figure 4 cluster plots are based. These plots show a very good match of co-ancestry patterns in the two African admixed ACB and ASW populations. The correlation analysis and r^2^ values aligned below the cluster plots in Figure 4 indicate high levels of correlation between Origins and BT co-ancestry proportion estimates for ACB and ASW, notably for the AFR proportions (although they are combined with AMR co-ancestry in these assessments). The EUR co-ancestry proportions tend to be slightly underestimated by BT SNPs or go undetected when very small. The four admixed populations with AMR co-ancestry are arranged in descending overall proportions of this co-ancestry and all show the same tendency for BT SNPs to underestimate the EUR proportions, but the PEL, MXL and CLM patterns still show a good match and relatively high r^2^ values for both main EUR and AMR co-ancestries. When three co-ancestries are detected, the BT SNPs have less precision to detect AMR and AFR co-ancestry at levels below 25%, particularly evident in the PUR non-EUR co-ancestry proportion estimates. Overall, the patterns match very well for simple two-way co-ancestry across most ratios, with BT only failing to detect very minor co-ancestry when it falls below 5%. The match of patterns only tends to reduce when 3-way co-ancestry is more prevalent, notably when the two minor co-ancestries are both below 10%. BT SNPs can detect two-way co-ancestry using STRUCTURE with sufficient accuracy for proportions to be reliably reported down to about 10% minor co-ancestry, although when three-way co-ancestry is detected, more caution would be required in reporting minor ratios below this 10% threshold and the lack of accuracy in such cases should be highlighted in the report. 

#### 3.5.2. Using GDA as a Simple Snipper-Based Evaluation of Admixture

From the STRUCTURE analysis of the BT SNPs, co-ancestry proportion estimates were compared to equivalent proportions provided by GDA in *Snipper*. This intended to test the efficiency of GDA to detect admixture and alert the user to the need to perform STRUCTURE analysis, if this initial analysis of the sample data does not suggest straightforward unadmixed ancestry. Figure S3A shows cluster plots for the STRUCTURE run and for the co-ancestry proportions based on GDA values, with r^2^ plots below arranged as in Figure 4. The r^2^ values indicate a good match between each approach, but GDA underestimates the EUR co-ancestry to a significant degree in ACB and ASW, to the point where GDA did not detect this co-ancestry in a large proportion of individuals, and it was underestimated by more than 50% of the true value (from Human Origins SNP array data) in most of the others. The trend of GDA underestimating European co-ancestry continues across all the PEL, MXL, CLM and PUR samples, where additionally, AFR co-ancestry is consistently overestimated in each population. The EUR underestimation is partly explained by detection of significant proportions of EAS co-ancestry by GDA, likely to be falsely created by a close relationship between AMR and EAS population groups, which STRUCTURE differentiates more efficiently as distinct genetic clusters compared to GDA’s measure of distance between EUR and both AMR and EAS. 

#### 3.5.3. Comparisons of 10-Percentile Co-Ancestry Ratio Patterns

A final visual comparison of co-ancestry patterns was made by plotting the mean co-ancestry ratios per 10-percentile group of individuals to smooth the data and allow easier assessment of trends in both the use of the small-scale BT ancestry panel to detect co-ancestry and GDA as an initial measure of the presence of admixture. Mean STRUCTURE co-ancestry proportions obtained with Origins SNPs and BT SNPs, plus GDA proportions, are plotted in Figure S3B. When admixture patterns are summarised into percentile groups, it is much easier to see the very close match in estimated co-ancestry ratios between Origins and BT SNP-based STRUCTURE runs. This is particularly evident in the 10th ASW percentile with a three-way co-ancestry pattern, and the 10th PEL percentile with a fourth EAS co-ancestry. However, co-ancestry proportions below 10% regularly fail to be detected by BT (1st ACB percentile, 3rd–5th PEL, 1st–2nd MXL, 9th–10th CLM percentiles). Therefore, it remains prudent to recognise that BT will not consistently detect co-ancestry ratios ≤10%. The presence of EAS co-ancestry at very small proportions in many of the MXL-CLM-PUR BT percentile groups indicates this component should be ignored when it is less than 10% and AMR co-ancestry is present above this ratio at the same time. The percentile group patterns in GDA emphasise that it is not an efficient technique for detecting admixture accurately when co-ancestral ratios are below 20%, so it does not provide an accurate measure of individuals with these proportions and STRUCTURE would be required in all cases, particularly as the bulk of the PEL-MXL-CLM percentile groups show spurious EAS co-ancestry ratios above this threshold value).

### 3.6. Audit of Additional Variation in BT SNPs from the GnomAD 3.1 Database

File S5 lists the co-amplified flanking SNPs and extra third-substitution alleles amongst both ancestry and appearance binary BT SNPs detected in the gnomAD 3.1 database. Twelve ancestry SNPs and three appearance SNPs had a third substitution allele, which reached the highest allele frequency of only 0.0002 in rs2302013 and rs917115 (both in East Asians). Ancestry SNP rs1063677 and appearance SNP rs12821256 showed all four substitution alleles, with the highest frequency for allele T of 0.0005 in Europeans in the GC SNP rs1063677.

The power of gnomAD to detect low frequency variants across the genome enabled sequence screens that indicated a large number of flanking SNPs within 25 nucleotides of the target site of many BT SNPs. Amongst the 115 ancestry SNPs, 49 had flanking SNPs, while in the 41 appearance (EVC) SNPs, there were 23, notably markers targeted in the SNP-rich MC1R gene. Four ancestry SNPs showed informative combinations of alleles in close proximity which show unlinked allele frequencies between the two loci—in other words, forming population-informative 2-SNP microhaplotypes (i.e., diplotypes). These four are detailed in Figure S4. The Chr-3 pair rs1169671-rs76444263 (target SNP in bold), Chr-8 pair rs75064826-rs13280988 and Chr-19 pair rs2337025-rs2337024 all show SAS-informative diplotypes, while the Chr-20 pair rs4809578-rs310644 has a range of diplotypes, which are most differentiating for AFR and EAS population groups. Microhaplotype rs1169671-rs76444263 combines a tri-allelic and binary SNP, but the latter has low levels of polymorphism so is less informative than the other binary SNP pairs. Diplotype SNP allele combinations can be analysed in *Snipper* by assigning A, C, G, T allele nomenclature to the alphabetic arrangement of diplotypes, which, for the above binary SNP pairs, would be A=AA, C=AG, G=CA, T=CG combinations; A=AA, C=AG, G=GA, T=GG combinations; and A=AC, C=AT, G=TC, T=TT combinations, respectively. While the phase of each SNP pair’s alleles needs to be detected from the sequence data to do this, it indicates the potential extra variant information, which is accessible from MPS analysis of forensic SNPs.

## 4. Concluding Remarks

The primary aim of the VISAGE Consortium of creating an all-in-one forensic intelligence tool based on MPS technology requires the development of a full analytical pipeline. This should include population reference data and predictive algorithms for the inference of age, ancestry and appearance characteristics associated with the genotyped SNPs, plus an optimum sequencing protocol which is applicable to the two main forensic MPS platforms in current use. The strategy of starting with a prototype tool of simple age predictors in one multiplex, and established pigmentation phenotype and continental ancestry predictors in the second, allowed us to build a strong foundation on which to develop the more ambitious VISAGE Enhanced Tool (ET). ET represents substantial expansion of all three ‘AAA’ predictive systems in the same parallel MPS multiplex setup as BT (i.e., separate DNA workflows dedicated to methylation analysis and SNP genotyping). The ancestry panel of BT therefore took the best ancestry informative SNPs from several well-established forensic ancestry panels, with particular regard to known MPS sequence reliability as well as optimum ancestry informativeness. For this reason, BT has provided very good sequencing performance with forensic DNA [29,37], and enables ancestry inferences to be made for populations of continental and South Asian origin with the highest levels of statistical likelihood.

Although we dedicated considerable resources to genotyping BT SNPs in populations addressing several geographic gaps in coverage left by large-scale genomic sequencing projects, many global regions remain to be characterised with new studies. The dynamic system in *Snipper*, i.e., compiling the largest possible array of population data and allowing the end-user to select which samples extend or modify the standardised reference set, provides flexibility for statistical analyses of BT SNP data. It also allows new population data to be uploaded to the webpages holding reference sets (http://mathgene.usc.es/snipper/forensic_mps_aims.html, accessed on 20 August 2021) and the additional populations shown in File S1. Although genetic cluster analyses using STRUCTURE can be challenging for forensic practitioners analysing population data for the first time, in our view, it is the best way to detect and analyse individual co-ancestry in persons with admixed backgrounds. The comparisons of GDA and STRUCTURE made in this study indicate that GDA is not accurate enough to evaluate low level co-ancestry when admixture patterns are complex and an individual has more than two co-ancestries. However, when the BT ancestry SNPs are compared to the much larger panel of Human Origins SNPs, the level of detail obtained using STRUCTURE is well matched and indicates BT SNPs are informative enough to accurately detect co-ancestral components present above 10% proportions.

Lastly, the care taken in selecting the BT ancestry SNPs based on their recorded reliability and sensitivity in previous MPS-based forensic ancestry panels [8,12,18,20,23] has helped to ensure predictable performance going forward with evaluation of both forensic MPS platforms. This extends to the sequencing chemistry, library preparation and sequence read balance (between strands, between alleles, within loci, between loci) observed so far in the forensic validations the VISAGE Consortium has made following SWGDAM guidelines for newly developed forensic DNA techniques (AmpliSeq chemistry [29], PowerSeq chemistry [37], and ForenSeq chemistry with a manuscript in submission). These same principals have been applied to the development of the ET ancestry panel, providing the means to increase the overall multiplex size of the tool and extending the range of populations among which ET ancestry SNPs can differentiate.

## Figures and Tables

**Table 1:** Commonality between SNPs selected for the BT ancestry panel and established forensic ancestry panels: Kiddlab 56; Thermo Fisher Precision ID Ancestry Panel (PIAP); Euroforgen Global AIMs (gAIMs); the LACE panel; the NAME panel; Eurasiaplex; and Shriver et al. US admixture mapping panel [24]. The two markers lower right are BT appearance SNPs previously selected as AIMs.

No.	Pop.	SNP	KK/PIAP	gAIMs	LACE	Other	No.	Pop.	SNP	KK/PIAP	gAIMs	LACE	Other	No.	Pop.	SNP	KK/PIAP	gAIMs	LACE	Other
1	AFR	rs10497191	Kiddlab	-	LACE		1	AME	rs10012227	-	gAIMs	-		1	Eurasia	rs1495085	-	-	-	NAME
2	AFR	rs1197062	-	gAIMs	LACE		2	AME	rs10483251	-	gAIMs	LACE		2	Eurasia	rs1757928	-	-	-	NAME
3	AFR	rs1369290	-	gAIMs	-		3	AME	rs12130799	PIAP	-	-		3	Eurasia	rs2337024	-	-	-	NAME
4	AFR	rs2789823	-	gAIMs	-		4	AME	rs12498138	Kiddlab	gAIMs	-		4	Eurasia	rs6989963	-	-	-	NAME
5	AFR	rs2814778	Kiddlab	gAIMs	-		5	AME	rs12629908	PIAP	-	-		5	Eurasia	rs6990312	Kiddlab	-	-	
6	AFR	rs310644	Kiddlab	gAIMs	-		6	AME	rs1452501	-	gAIMs	LACE		6	Eurasia	rs7148809	-	-	-	NAME
7	AFR	rs4737753	-	-	-	NAME	7	AME	rs1557553	-	gAIMs	LACE		7	Eurasia	rs12203115	-	-	-	NAME
1	EUR	rs11778591	-	gAIMs	LACE		8	AME	rs17130385	-	gAIMs	LACE		8	Eurasia	rs2227203	-	-	-	NAME
2	EUR	rs12142199	-	gAIMs	-		9	AME	rs17359176	-	gAIMs	LACE		9	Eurasia	rs39897	-	-	-	Eurasiaplex
3	EUR	rs12913832	Kiddlab	gAIMs	-		10	AME	rs174570	Kiddlab	gAIMs	LACE		10	Eurasia	rs4308478	-	-	-	NAME
4	EUR	rs1426654	Kiddlab	gAIMs	-		11	AME	rs2302013	-	gAIMs	-		11	Eurasia	rs7570971	-	-	-	NAME
5	EUR	rs16891982	Kiddlab	gAIMs	-		12	AME	rs2471552	-	gAIMs	-		12	Eurasia	rs984038	-	-	-	NAME
6	EUR	rs2715883	-	gAIMs	-		13	AME	rs3737576	Kiddlab	-	-		1	SAS	rs1040934	-	-	-	Shriver
7	EUR	rs3759171	-	gAIMs	LACE		14	AME	rs4792928	-	gAIMs	-		2	SAS	rs1063677	-	-	-	Shriver
8	EUR	rs705308	PIAP	-	-		15	AME	rs5757362	-	-	LACE		3	SAS	rs10764919	-	-	LACE	
9	EUR	rs7084970	-	gAIMs	-		16	AME	rs8137373	-	gAIMs	-		4	SAS	rs10962599	-	-	-	Eurasiaplex
10	EUR	rs7531501	-	gAIMs	-		17	AME	rs870347	Kiddlab	-	-		5	SAS	rs13267318	-	-	-	Shriver
11	EUR	rs8072587	-	gAIMs	-		1	EAS	rs10079352	-	gAIMs	LACE		6	SAS	rs13280988	-	-	LACE	
12	EUR	rs820371	-	gAIMs	LACE		2	EAS	rs1229984	Kiddlab	gAIMs	-		7	SAS	rs17625895	-	-	-	Eurasiaplex
13	EUR	rs862500	-	gAIMs	LACE		3	EAS	rs12594144	-	gAIMs	-		8	SAS	rs1796048	-	-	-	Shriver
14	EUR	rs917115	Kiddlab	gAIMs	-		4	EAS	rs1371048	-	gAIMs	-		9	SAS	rs1924381	-	gAIMs	LACE	
15	EUR	rs9522149	Kiddlab	gAIMs	-		5	EAS	rs17822931	-	gAIMs	-		10	SAS	rs2026999	-	-	-	Shriver
1	OCE	rs10149275	-	gAIMs	-		6	EAS	rs1834619	Kiddlab	gAIMs	-		11	SAS	rs2196051	Kiddlab	-	-	Eurasiaplex
2	OCE	rs16830500	-	gAIMs	-		7	EAS	rs2180052	-	gAIMs	-		12	SAS	rs2238151	Kiddlab	-	-	
3	OCE	rs2139931	-	gAIMs	-		8	EAS	rs3827760	Kiddlab	gAIMs	-		13	SAS	rs2269793	PIAP	-	-	
4	OCE	rs2274636	-	gAIMs	-		9	EAS	rs434504	-	gAIMs	-		14	SAS	rs2472304	-	-	-	Eurasiaplex
5	OCE	rs26951	-	gAIMs	-		10	EAS	rs459920	Kiddlab	-	-		15	SAS	rs2503770	-	gAIMs	LACE	
6	OCE	rs3751050	-	gAIMs	-		11	EAS	rs4657449	-	gAIMs	-		16	SAS	rs26247	-	-	-	Shriver
7	OCE	rs3804030	-	gAIMs	-		12	EAS	rs4781011	PIAP	-	-		17	SAS	rs3844336	-	-	-	Shriver
8	OCE	rs4391951	-	gAIMs	-		13	EAS	rs4918664	Kiddlab	gAIMs	-		18	SAS	rs7080350	-	-	LACE	
9	OCE	rs4959270	-	x	-	Pacifiplex *	14	EAS	rs4935501	-	gAIMs	-		19	SAS	rs7568054	-	-	LACE	
10	OCE	rs6054465	-	gAIMs	-		15	EAS	rs7226659	Kiddlab	-	-		20	SAS	rs756913	-	-	-	Eurasiaplex
11	OCE	rs715605	-	gAIMs	-		16	EAS	rs8104441	-	gAIMs	-								
12	OCE	rs9908046	-	gAIMs	-									MYH15 º	EAS	rs6437783	-	gAIMs	-	-
13	OCE	rs9934011	-	gAIMs	-									OCA2 º	EAS	rs1800414	Kiddlab	-	-	-

* A single SNP developed for the Pacifiplex panel was not incorporated into gAIMs. º Gene locations of the two BT appearance SNPs previously selected for other ancestry panels.

## Data Availability

All data generated in these studies, except the cross-validation likelihoods, are included in the Files.

## Supplementary Materials

The following are available online at https://www.mdpi.com/article/10.3390/genes12081284/s1, Files S1: Allele frequency estimates in seven population groups for the 115 ancestry SNPs of BT; S2A: Snipper Reference Grid; S2B: 1KG Unadmixed Populations; S2C: 1KG Admixed Populations; S2D: HGDP-CEPH Populations; S2E: VISAGE In-house Populations; S2F: SGDP Test Samples; S2G: EGDP Test Samples, S3A: Genotype comparisons of 1000 Genomes HGDP-CEPH data (red) vs VISAGE in-house HGDP-CEPH data (black); S3B: Genotype comparisons of 1000 Genomes Phase III low-coverage data based on 2-3X sequence coverage (black) vs. 1000 Genomes high coverage data based on New York Genome Centreߣs 30X resequencing of the same samples (blue); S4: Genetic cluster membership proportions from the four major contributing populations to American admixture with 572K SNPs of Human Origins array analyses using ADMIXTURE vs 115 BT SNPs using STRUCTURE, for 1511 unadmixed and 504 admixed 1000 Genomes samples; S5: Screen of all 153 BT SNPs of gnomAD genome database information for current GRCh37-38 positions, gene identifiers, third alleles and high frequency flanking SNPs (present on the same sequence strand as the target site within +/− 25 nucleotides). Figures S1A: 7 African-informative SNPs; S1B: 15 European-informative SNPs; S1C: 16 East Asian-informative SNPs; S1D: 20 South Asian-informative SNPs; S1E: 13 Oceanian-informative SNPs; S1F: 17 American-informative SNPs; S1G: 12 Eurasian divergent SNPs, S2.1: Bio-geographical ancestry analysis based on the six reference populations (1–6, summarised in Figure 1) plus unadmixed (8–11, 14–25) and admixed (12–13, 26–29) populations available from 1000 Genomes 30X high coverage data (1KG-30); S2.2: Bio-geographical ancestry analysis based on the six reference populations presented in Figure 1, plus populations available from HGDP-CEPH Whole Genome Sequencing Data; S2.3: Bio-geographical ancestry analysis based on the six reference populations presented in Figure 1, plus populations available from in-house genotyping with the VISAGE BT tool and SGDP data; S2.4: Bio-geographical ancestry analysis based on the six reference populations presented in Figure 1, plus populations available from EGDP data, with ~85% (95/115 SNPs) profile completeness. S3.1: (A) Co-ancestry proportions sample-by-sample in 504 1000 Genomes admixed individuals based on STRUCTURE and GDA analyses. (B) Correlation analyses and r2 values comparing the combined African-American and European co-ancestry proportion estimates with both techniques. S3.2: Mean co-ancestry proportions summarised as 10-percentile columns (10 bins of 10% of each set of individuals), of varyingnumbers per bin: ACB: 10, ACB: 10, ASW: 6, PEL: 8, ACB: 10, MXL: 6, CLM: 9, PUR: 10. S4: Four BT ancestry SNP target sites and population informative flanking SNPs within 25 nucleotides (nt) on the 5’ or 3’ side. Table S1: Individual and cumulative population specific Divergence (PSD) values for the 115 ancestry SNPs arranged in descending order of PSD value per population.

## Appendix A

Centres and investigators of the VISible Attributes through GEnomics (VISAGE) Consortium, Website: http://www.visage-h2020.eu/ (accessed on 20 August 2021).

- Erasmus University Medical Center Rotterdam, Rotterdam, The Netherlands: Manfred Kayser, Vivian Kalamara, Arwin Ralf, Athina Vidaki
- Jagiellonian University, Krakow, Poland: Wojciech Branicki, Ewelina Pośpiech, Aleksandra Pisarek
- Universidade de Santiago de Compostela, Santiago de Compostela, Spain: Ángel Carracedo, Maria Victoria Lareu, Christopher Phillips, Ana Freire-Aradas, Ana Mosquera-Miguel, María de la Puente
- Medizinische Universität Innsbruck, Innsbruck, Austria: Walther Parson, Catarina Xavier, Antonia Heidegger, Harald Niederstätter
- Universität zu Köln, Cologne, Germany: Michael Nothnagel, Maria-Alexandra Katsara, Tarek Khellaf
- King’s College London, London, UK: Barbara Prainsack, Gabrielle Samuel
- Klinikum der Universität zu Köln, Cologne, Germany: Peter M. Schneider, Theresa E. Gross,

Jan Fleckhaus, Elaine Cheung.

- Bundeskriminalamt, Wiesbaden, Germany: Ingo Bastisch, Nathalie Schury, Jens Teodoridis,

Martina Unterländer

- Institut National de Police Scientifique, Lyon, France: François-Xavier Laurent, Caroline

Bouakaze, Yann Chantrel, Anna Delest, Clémence Hollard, Ayhan Ulus, Julien Vannier

- Netherlands Forensic Institute, The Hague, Netherlands: Titia Sijen, Kris van der Gaag,

Marina Ventayol-Garcia

- National Forensic Centre, Swedish Police Authority, Linköping, Sweden: Johannes Hedman,

Klara Junker, Maja Sidstedt

- Metropolitan Police Service, London, United Kingdom: Shazia Khan, Carole E. Ames, Andrew Revoir
- Centralne Laboratorium Kryminalistyczne Policji, Warsaw, Poland: Magdalena Spólnicka, Ewa Kartasinska, Anna Woźniak
