# Supplementary material for: Development and Evaluation of the Ancestry Informative Marker Panel of the VISAGE Basic Tool
Source: Genes (Basel). 2021 Aug 22;12(8):1284. doi: 10.3390/genes12081284 (PMC8391248; doi:10.3390/genes12081284)

Supplementary Fig. S1.A

7 African-informative SNPs

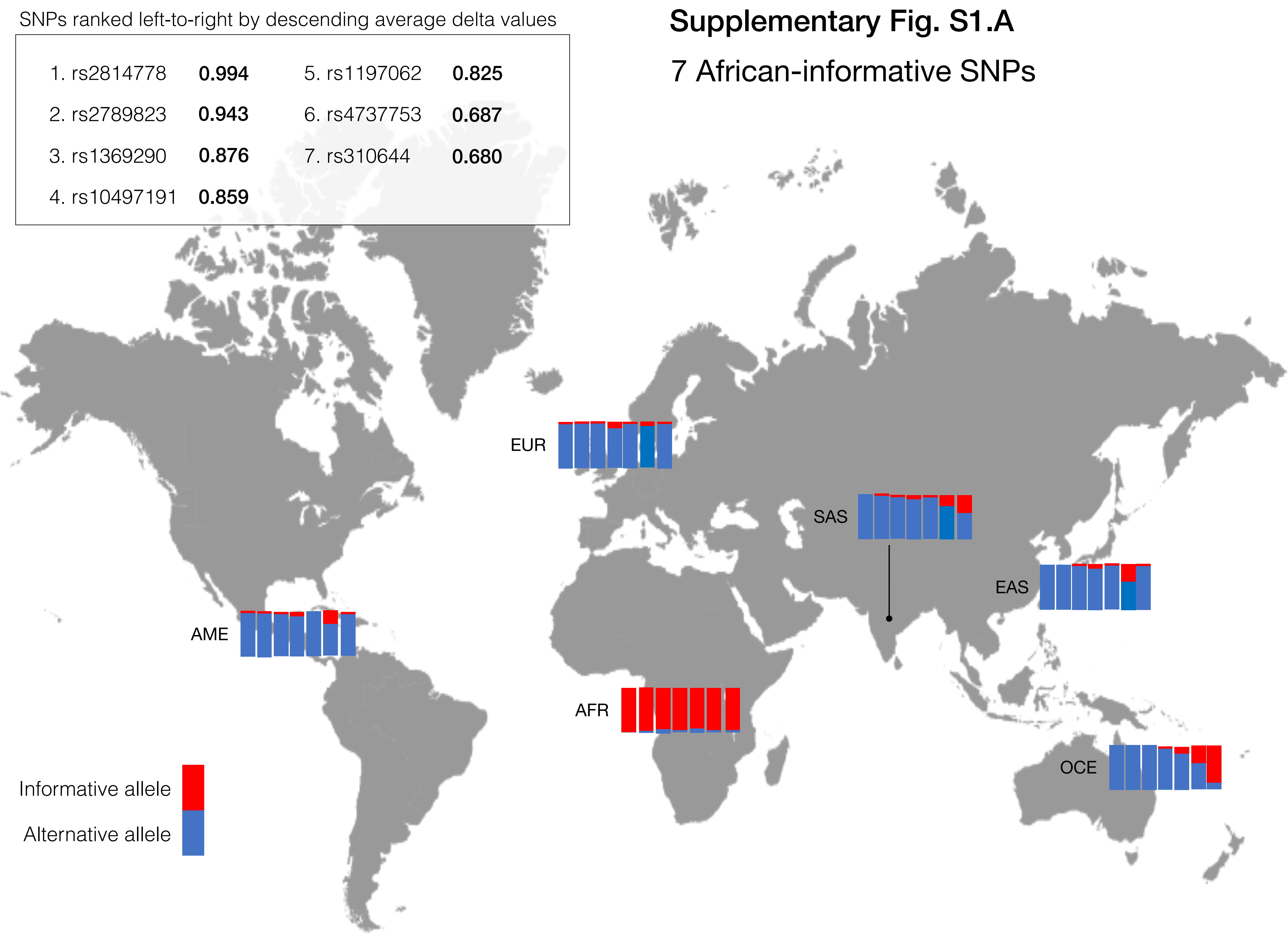

Supplementary Fig. S1.B

15 European-informative SNPs

SNPs ranked left-to-right by descending average delta values

1. rs1426654	**0.958**	9. rs3759171	**0.670**
2. rs16891982	**0.921**	10. rs2715883	**0.660**
3. rs12142199	**0.771**	11. rs917115	**0.612**
4. rs8072587	**0.740**	12. rs12913832	**0.607**
5. rs9522149	**0.736**	13. rs7084970	**0.595**
6. rs7531501	**0.729**	14. rs862500	**0.497**
7. rs11778591	**0.727**	15. rs705308	**0.490**
8. rs820371	**0.675**		

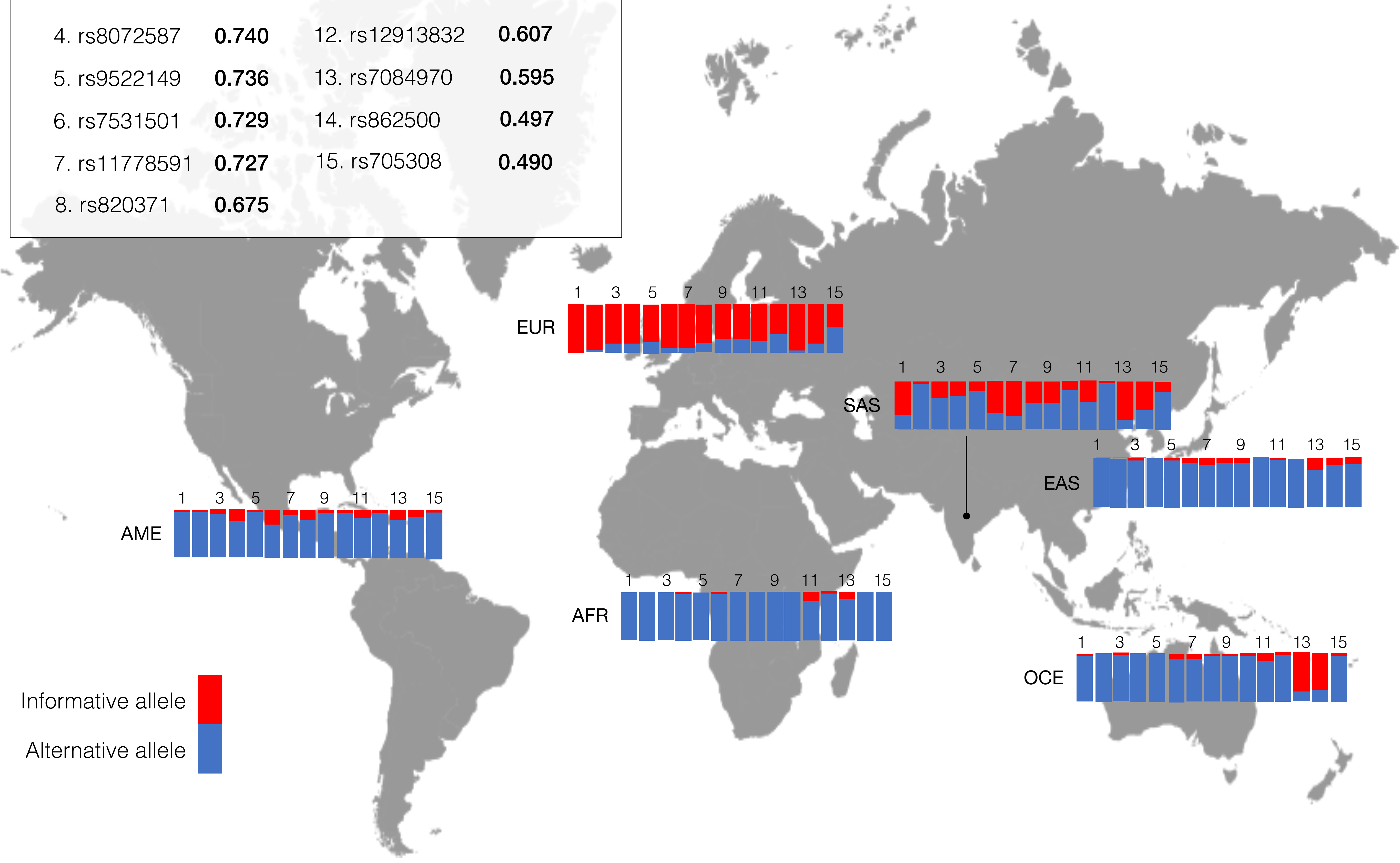

Supplementary Fig. S1.C

16 East Asian-informative SNPs

SNPs ranked left-to-right by descending average delta values

1. rs3827760	**0.850**	9. rs4935501	**0.561**
2. rs4918664	**0.754**	10. rs4657449	**0.553**
3. rs12594144	**0.702**	11. rs2180052	**0.518**
4. rs17822931	**0.686**	12. rs7226659	**0.498**
5. rs10079352	**0.676**	13. rs8104441	**0.487**
6. rs1229984	**0.669**	14. rs4781011	**0.472**
7. rs434504	**0.643**	15. rs1834619	**0.458**
8. rs1371048	**0.579**	16. rs459920	**0.207**

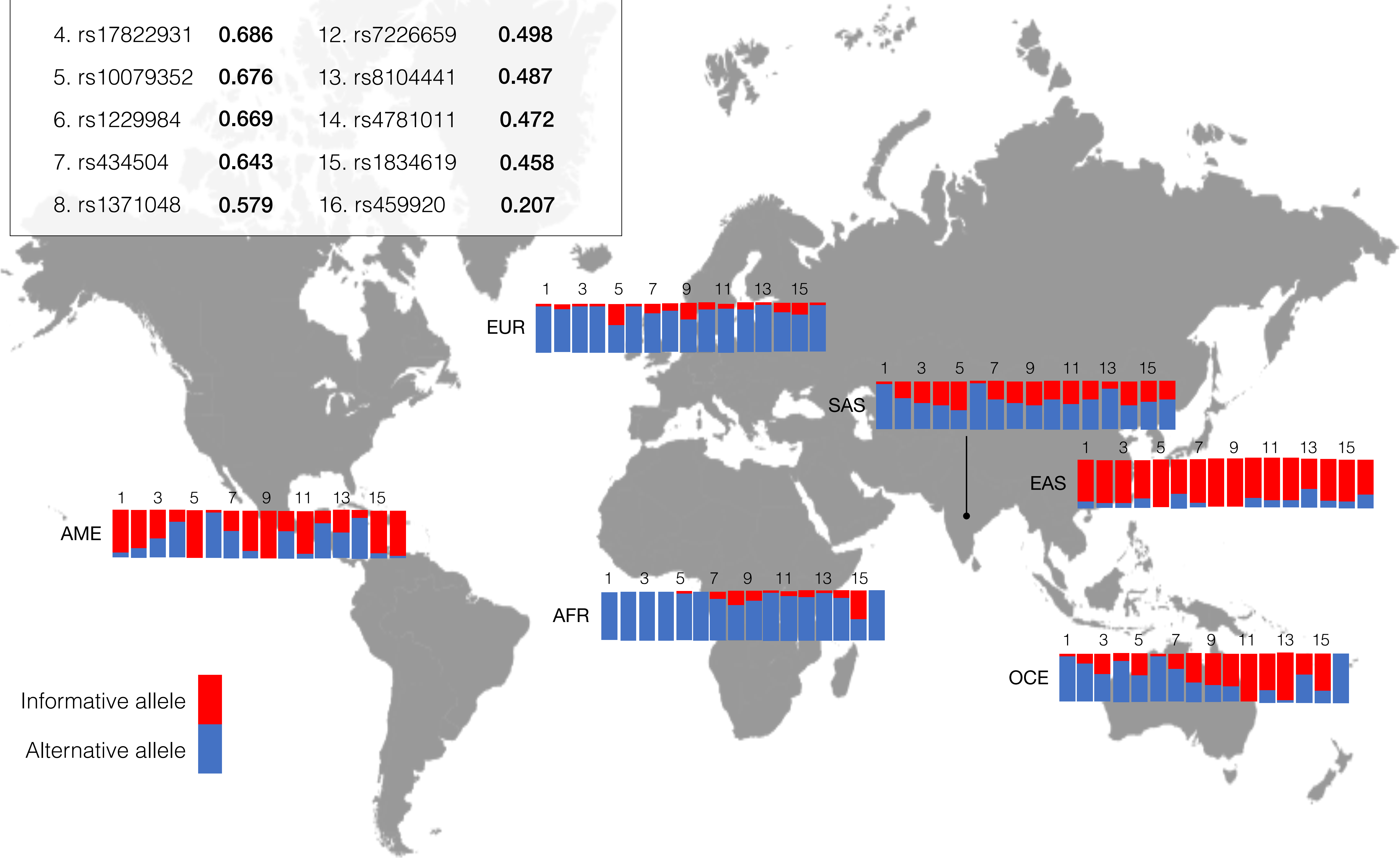

Supplementary Fig. S1.D

20 South Asian-informative SNPs

1. rs10962599	**0.552**	11. rs2026999	**0.398**
2. rs2196051	**0.523**	12. rs1924381	**0.392**
3. rs1796048	**0.497**	13. rs2238151	**0.376**
4. rs3844336	**0.496**	14. rs17625895	**0.370**
5. rs2472304	**0.442**	15. rs26247	**0.363**
6. rs1040934	**0.429**	16. rs7568054	**0.362**
7. rs1063677	**0.414**	17. rs756913	**0.355**
8. rs10764919	**0.409**	18. rs2503770	**0.348**
9. rs13280988	**0.409**	19. rs7080350	**0.342**
10. rs13267318	**0.400**	20. rs2269793	**0.197**

SNPs ranked left-to-right by descending **EUR-SAS** delta values

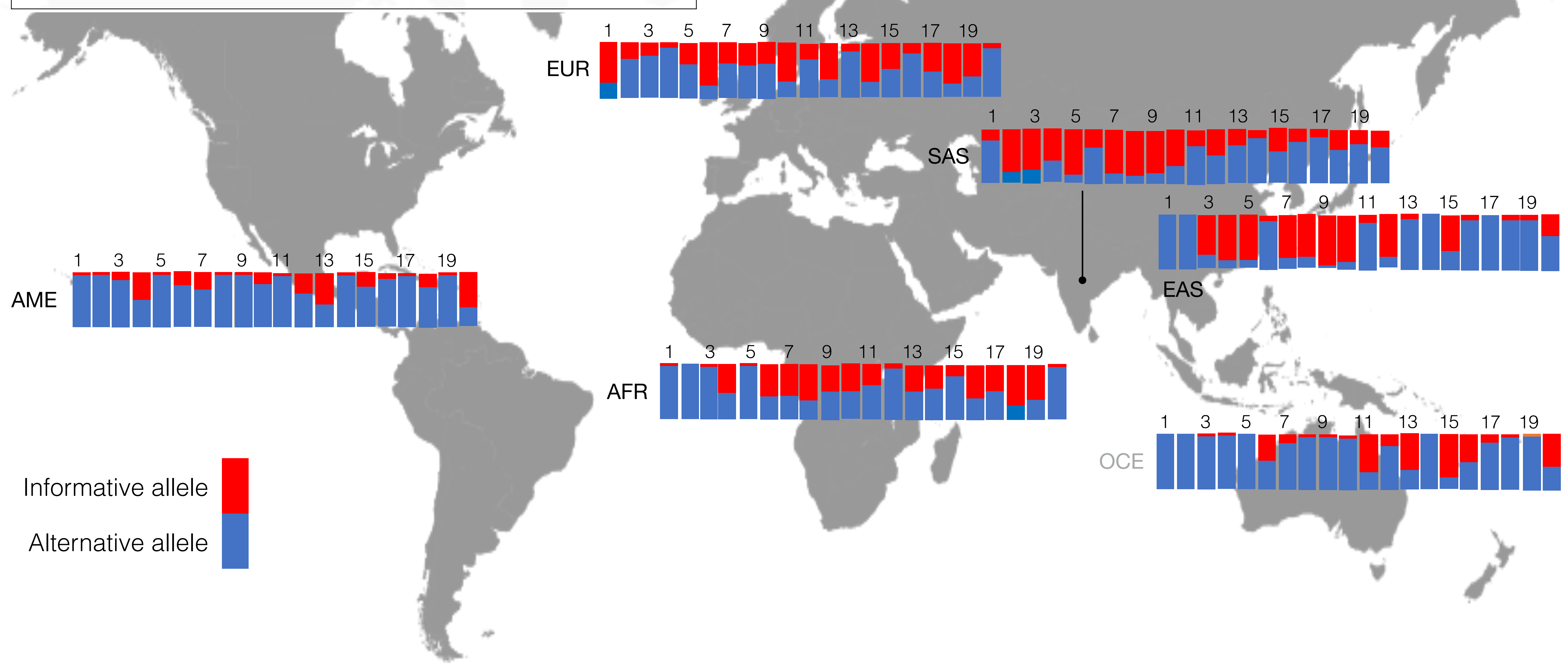

Supplementary Fig. S1.E

13 Oceanian-informative SNPs

SNPs ranked left-to-right by descending average delta values

1. rs9908046	**0.876**	8. rs26951	**0.762**
2. rs715605	**0.832**	9. rs4391951	**0.730**
3. rs3751050	**0.806**	10. rs2274636	**0.727**
4. rs2139931	**0.802**	11. rs10149275	**0.671**
5. rs6054465	**0.798**	12. rs9934011	**0.607**
6. rs3804030	**0.779**	13. rs4959270	**0.600**
7. rs16830500	**0.771**		

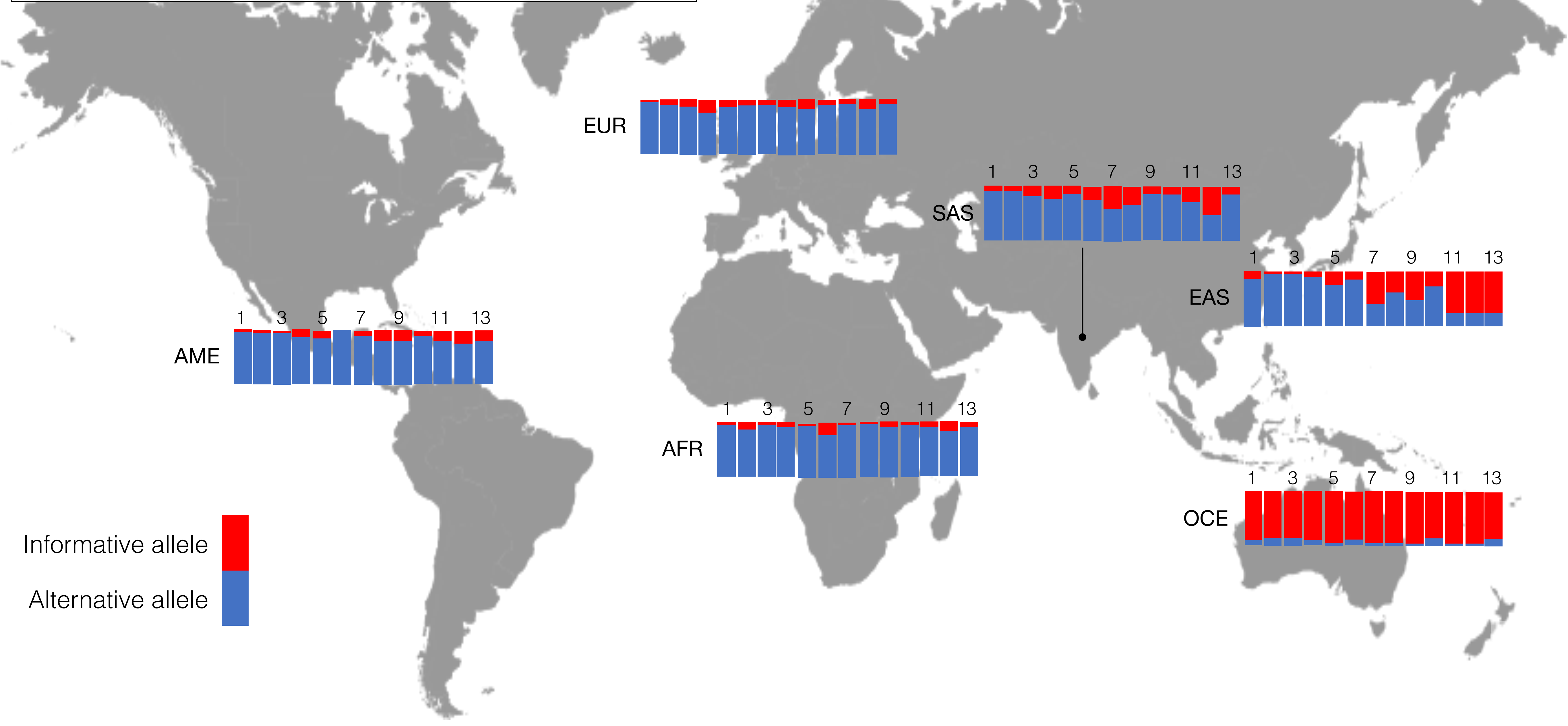

Supplementary Fig. S1.F

17 American-informative SNPs

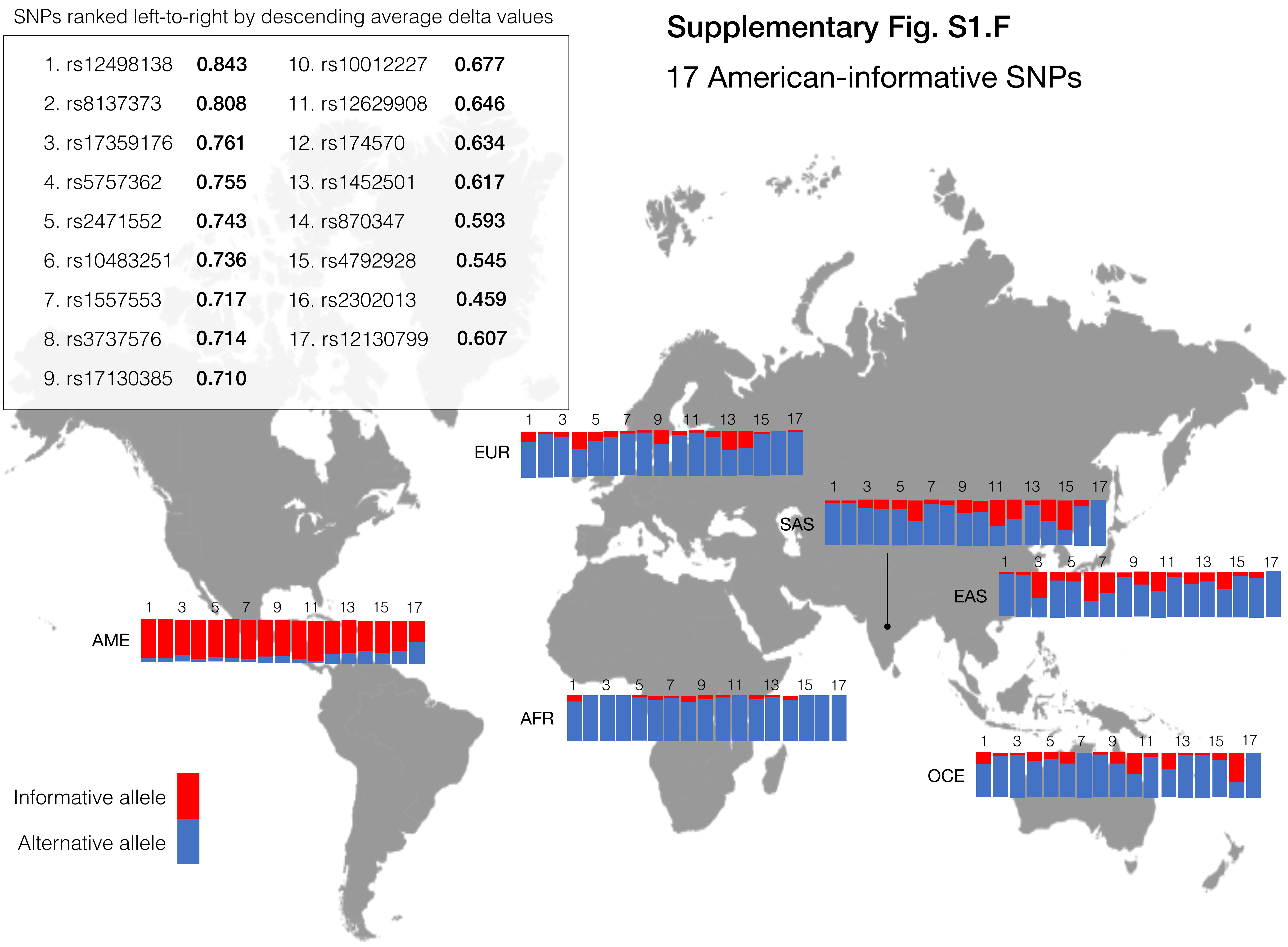

Supplementary Fig. S1.G

12 Eurasian divergent SNPs

SNPs ranked by descending **Eurasian (EUR-NAF-ME-SAS)** delta values

1. rs7570971	**0.452**	7. rs1757928	**0.603**
2. rs39897	**0.322**	8. rs1495085	**0.495**
3. rs984038	**0.291**	9. rs7148809	**0.479**
4. rs2227203	**0.291**	10. rs6989963	**0.462**
5. rs12203115	**0.214**	11. rs6990312	**0.181**
6. rs4308478	**0.214**	12. rs2337024	**0.149**

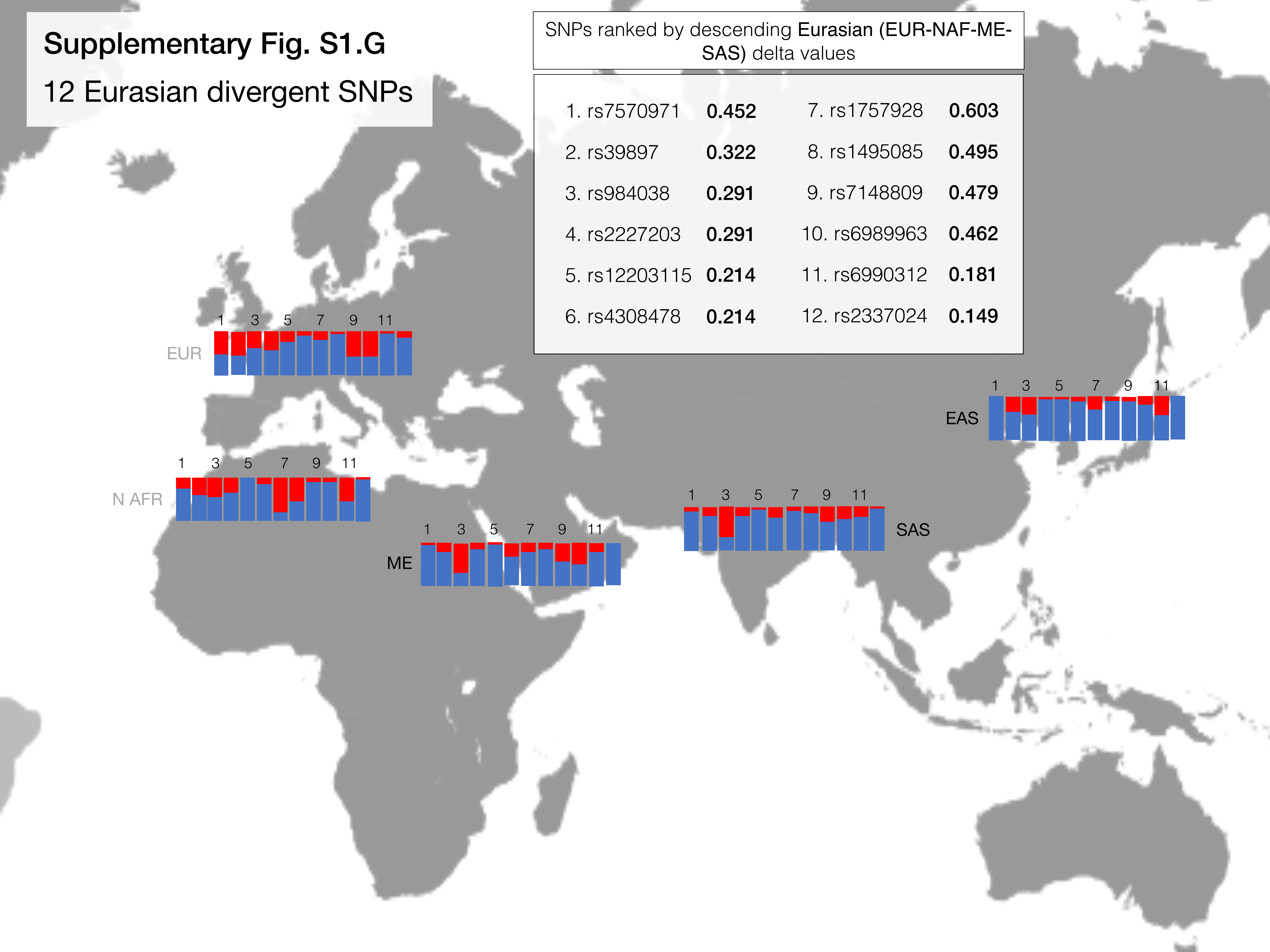

Supplement: Supplementary file 1 [file genes-12-01284-s001.zip › BT Supplementary Files and Figs/Supplementary Figure S1.pdf]
