# Supplementary material for: Development and Evaluation of the Ancestry Informative Marker Panel of the VISAGE Basic Tool
Source: Genes (Basel). 2021 Aug 22;12(8):1284. doi: 10.3390/genes12081284 (PMC8391248; doi:10.3390/genes12081284)

Supplementary Fig. S2.1. Bio-geographical ancestry analysis based on the six reference populations (1-6, summarised in Fig. 1) plus unadmixed (8-11, 14-25) and admixed (12-13, 26-29) populations available from 1000 Genomes 30X high coverage data (1KG-30). **(A)** STRUCTURE results of cluster membership proportions at K=6, with all non-reference populations classified against the reference dataset 1-6. For the STRUCTURE analysis, the inferred clusters are colored so the major proportion of each reference population matches the righthand legend. **(B)** Three dimensional MDS analysis showing coordinates 1 and 3 (top plot), 1 and 2 (bottom). **(C)** Neighbor Joining Tree (NJT) analysis. For the MDS and NJT plots, populations are colored according to the righthand legend. Note, AMR reference set 6 includes 18 PEL without co-ancestry and the remaining 67 admixed PEL are in set 26.

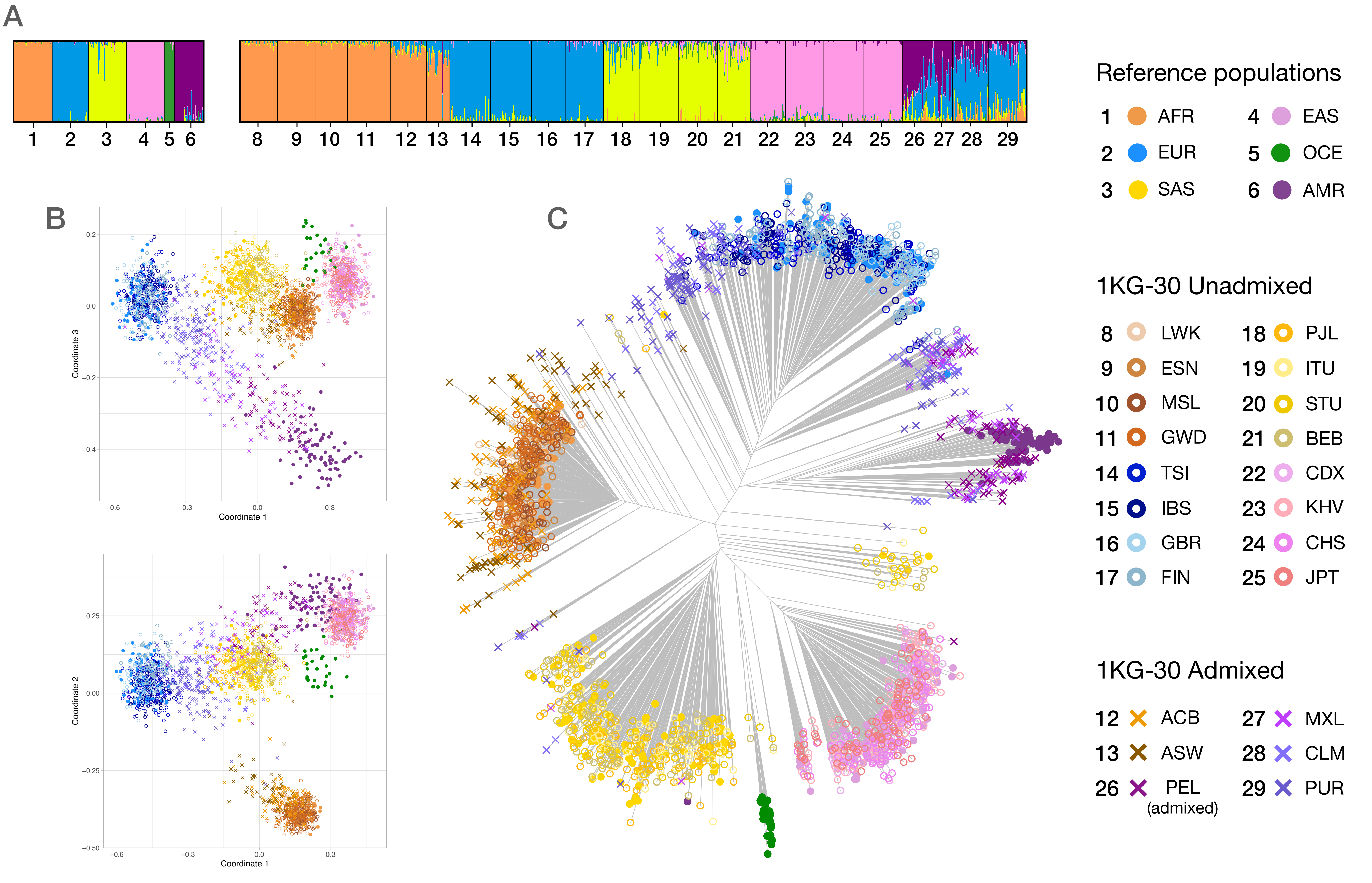

Supplementary Fig. S2.2. Bio-geographical ancestry analysis based on the six reference populations presented in **Fig. 1**, plus populations available from HGDP-CEPH Whole Genome Sequencing Data. **(A)** STRUCTURE results of ancestry proportions at K=6, non-reference populations were classified against the lefthand reference dataset 1-6. For the STRUCTURE analysis, the inferred clusters are colored so the major proportion of each reference population (1-6) matches the righthand legend. **(B)** Three dimensional MDS analysis showing coordinates 1 and 2 (up) and 1 and 3 (down). **(C)** Neighbor Joining Tree (NJT) analysis. For the MDS and NJT plots, populations are colored according to the righthand legend.

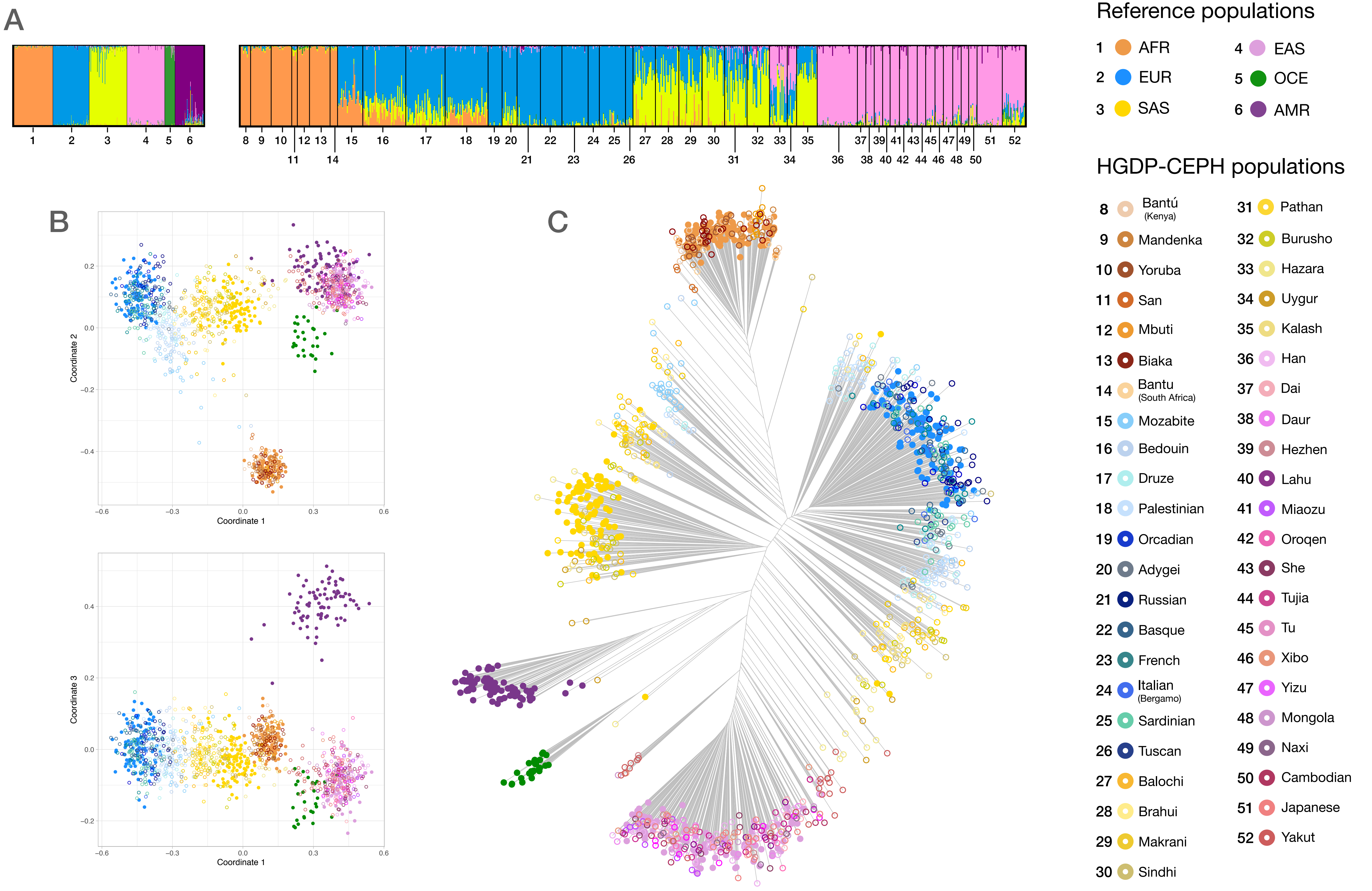

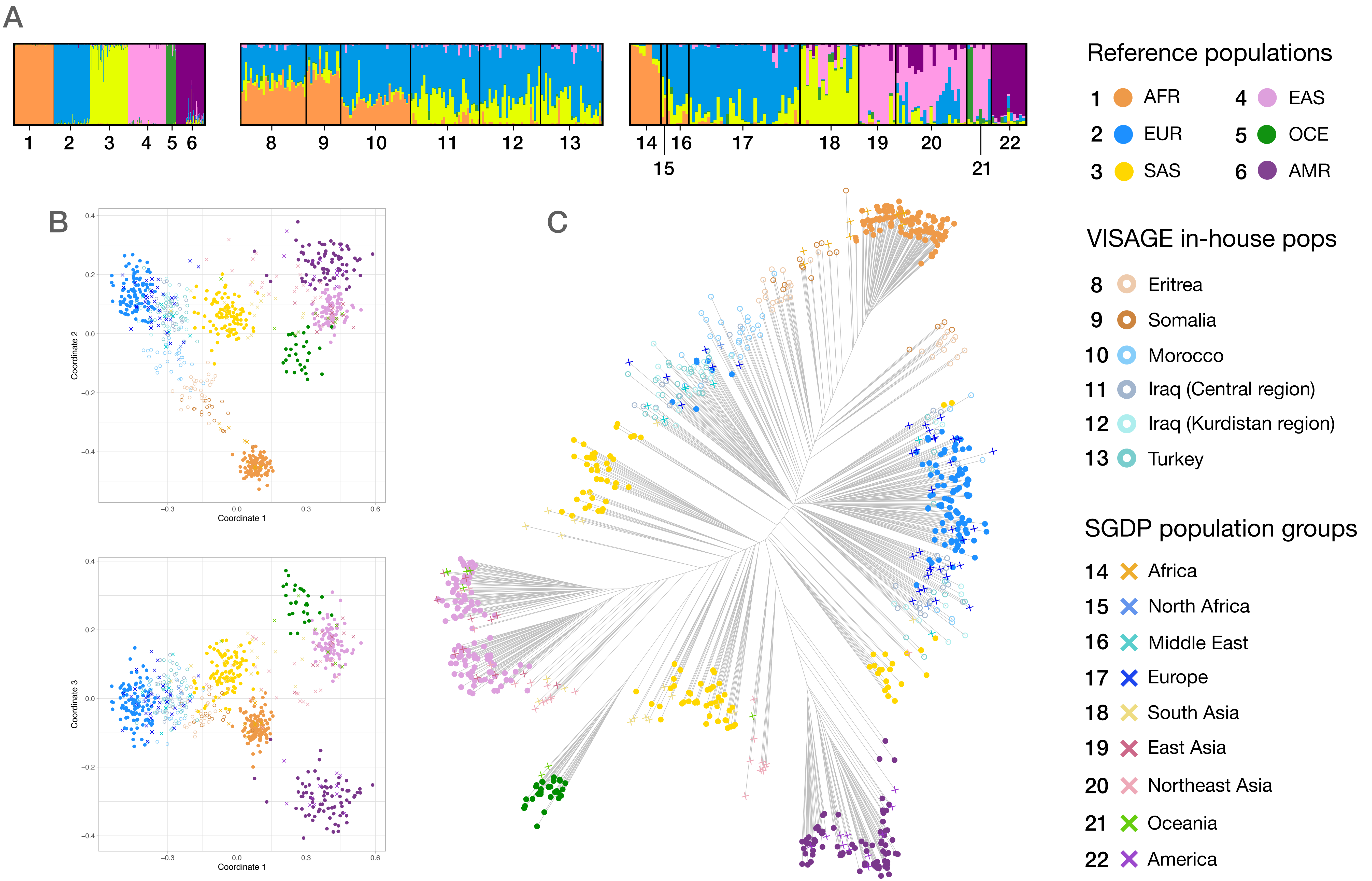

Supplementary Fig. S2.3. Bio-geographical ancestry analysis based on the six reference populations presented in **Fig. 1**, plus populations available from in-house genotyping with the VISAGE BT tool and SGDP data. **(A)** STRUCTURE results of cluster membership proportions at K=6, non-reference populations were classified against the lefthand reference dataset 1-6. For the STRUCTURE analysis, the inferred clusters are colored so the major proportion of each reference population matches the righthand legend. **(B)** Three dimensional MDS analysis showing coordinates 1 and 2 (top), 1 and 3 (bottom). **(C)** Neighbor Joining Tree (NJT) analysis. For the MDS and NJT plots, populations are colored according to the righthand legend.

Supplementary Fig. S2.4. Bio-geographical ancestry analysis based on the six reference populations presented in **Fig. 1**, plus populations available from EGDP data, with ~85% (95/115 SNPs) profile completeness. **(A)** STRUCTURE results of ancestry proportions at K=6, non-reference populations were classified against the reference dataset. For the STRUCTURE analysis, the inferred clusters are colored so the major proportion of each reference population (1-6) matches the righthand legend. **(B)** Three dimensional MDS analysis showing coordinates 1 and 2 (top plot), 1 and 3 (bottom). **(C)** Neighbor Joining Tree (NJT) analysis. For the MDS and NJT plots, populations are colored according to the righthand legend and the analysis is based on the 100 SNPs present in the EGDP data.

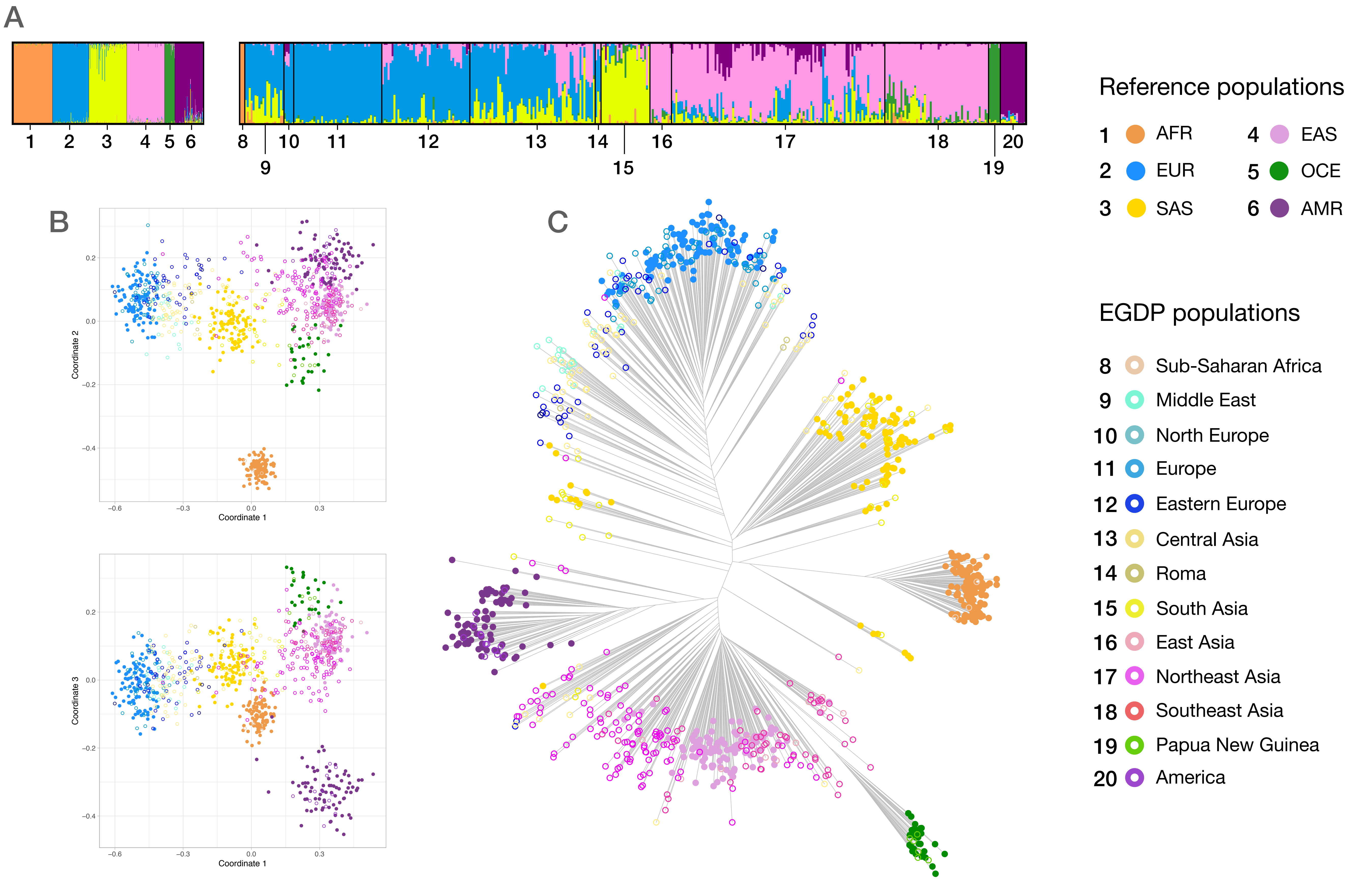

Supplement: Supplementary file 1 [file genes-12-01284-s001.zip › BT Supplementary Files and Figs/Supplementary Figure S2.pdf]
