# Supplementary material for: Development and Evaluation of the Ancestry Informative Marker Panel of the VISAGE Basic Tool
Source: Genes (Basel). 2021 Aug 22;12(8):1284. doi: 10.3390/genes12081284 (PMC8391248; doi:10.3390/genes12081284)

Supplementary Fig. S3.1 (A) Co-ancestry proportions sample-by-sample in 504 1000 Genomes admixed individuals based on STRUCTURE and GDA analyses. **(B)** Correlation analyses and r^2 values comparing the combined African-American and European co-ancestry proportion estimates with both techniques.

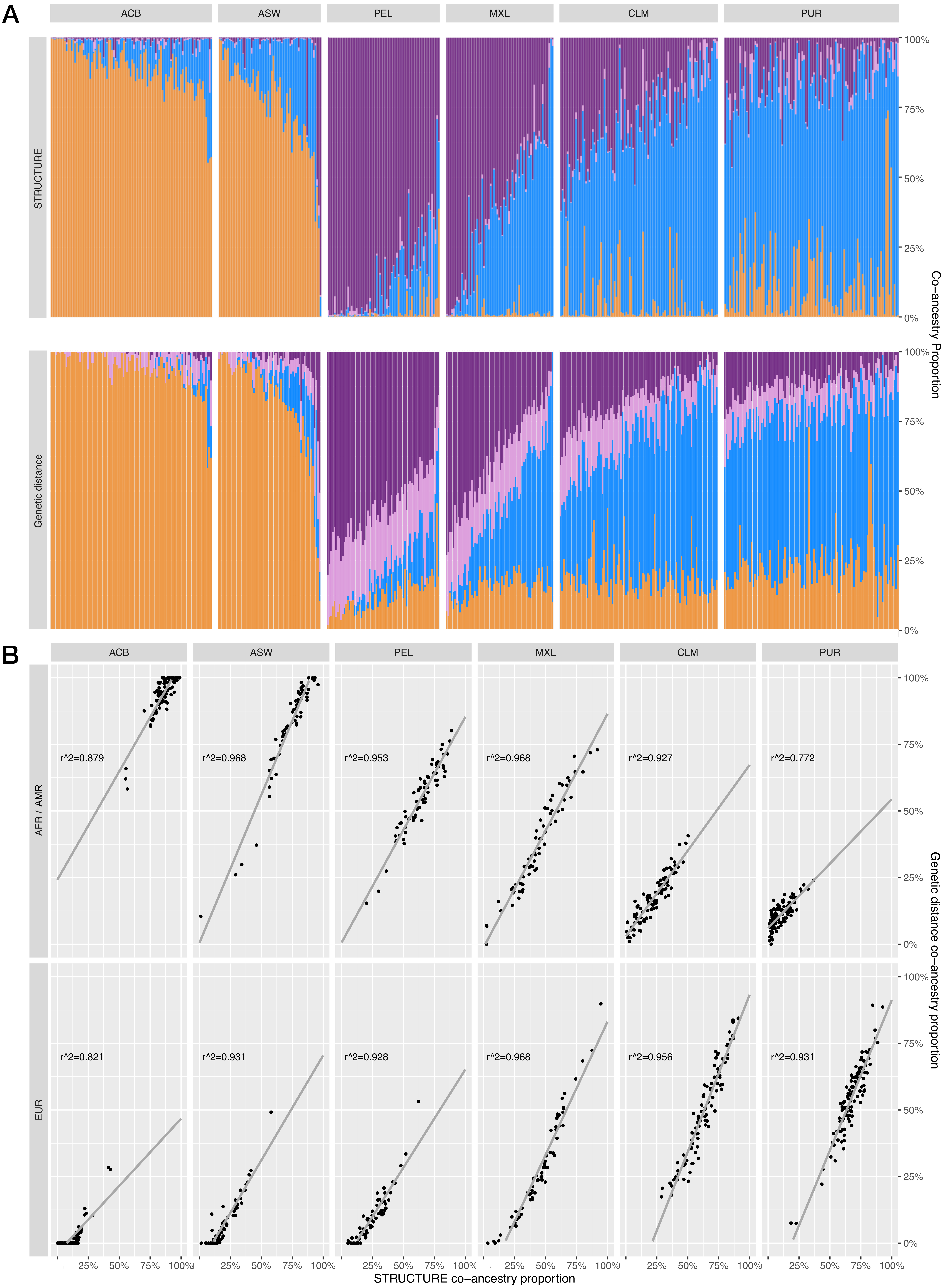

Supplementary Fig. S3.2 Mean co-ancestry proportions summarised as 10-percentile columns (10 bins of 10% of each set of individuals), of varying numbers per bin: ACB: 10, ACB: 10, ASW: 6, PEL: 8, ACB: 10, MXL: 6, CLM: 9, PUR: 10. **(A)** >572K Affymetrix Human Origins array SNPs analysed with STRUCTURE **(B)** 115 BT SNPs analysed with STRUCTURE **(C)** 115 BT SNPs analysed with GDA.

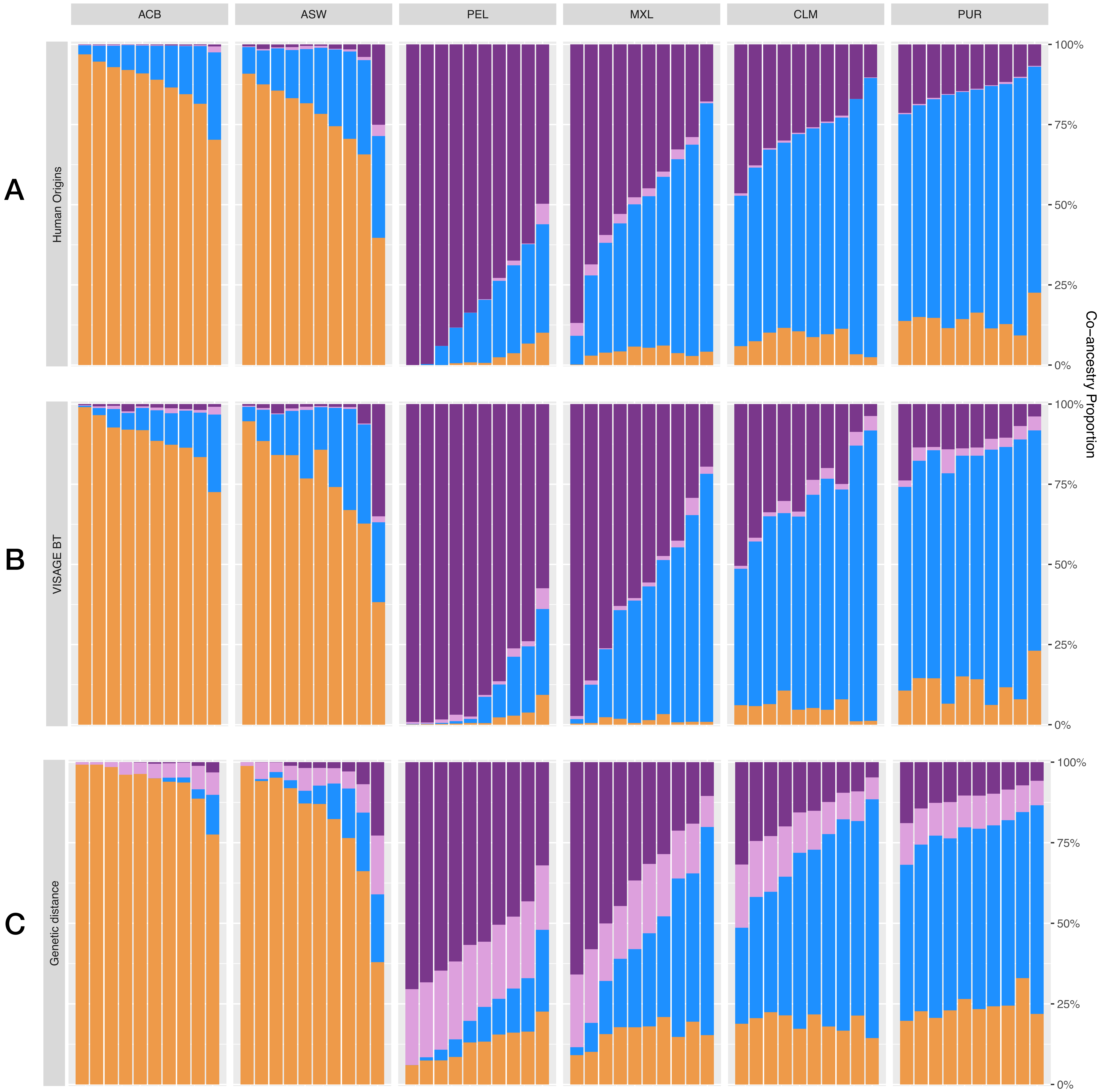

Supplement: Supplementary file 1 [file genes-12-01284-s001.zip › BT Supplementary Files and Figs/Supplementary Figure S3.pdf]
