# Supplementary material for: Development and Evaluation of the Ancestry Informative Marker Panel of the VISAGE Basic Tool
Source: Genes (Basel). 2021 Aug 22;12(8):1284. doi: 10.3390/genes12081284 (PMC8391248; doi:10.3390/genes12081284)

Supplementary Fig. S4. Four BT ancestry SNP target sites and population informative flanking SNPs within 25 nucleotides (nt) on the 5’ or 3’ side

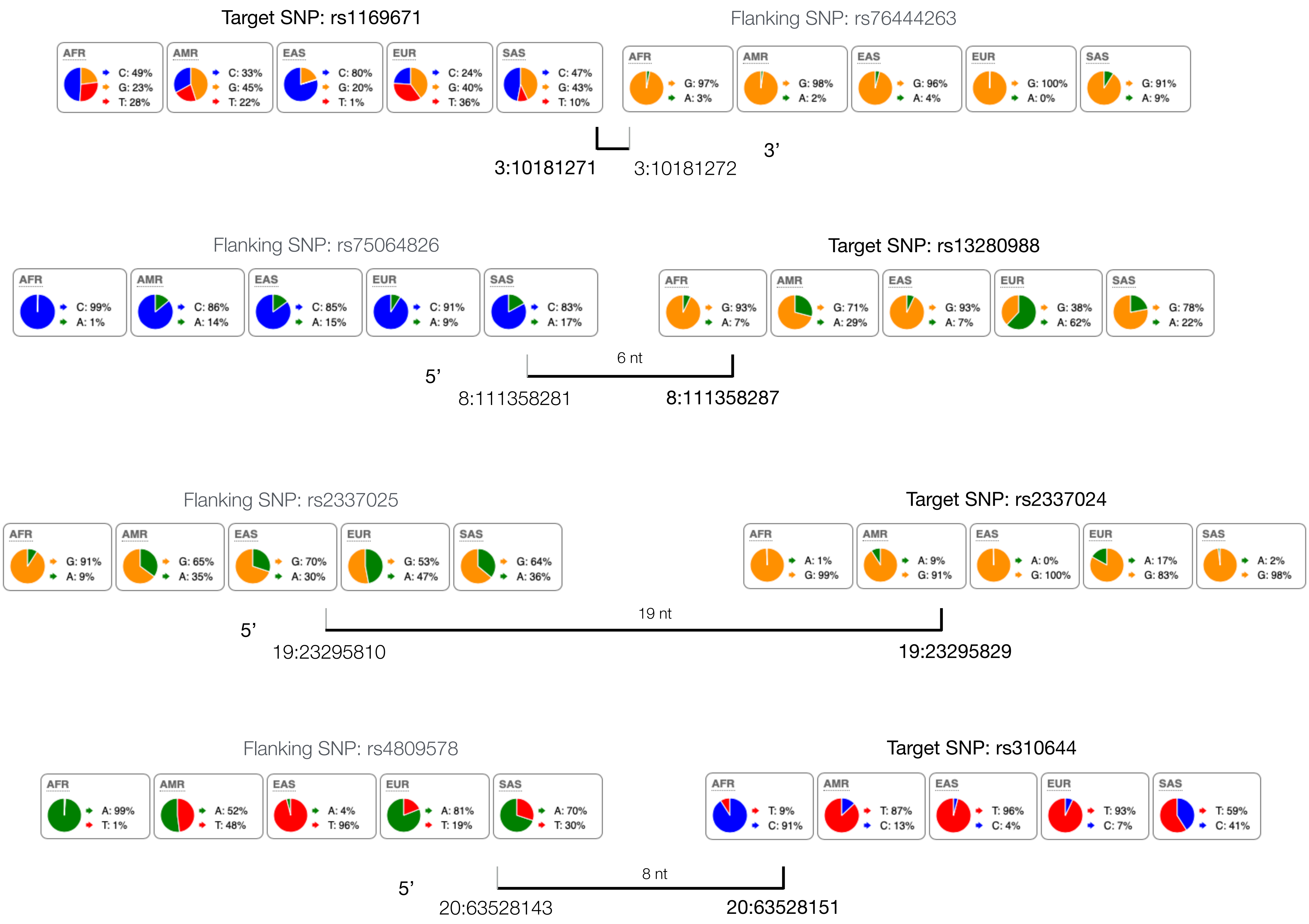

Supplement: Supplementary file 1 [file genes-12-01284-s001.zip › BT Supplementary Files and Figs/Supplementary Figure S4.pdf]
